# Shaping the biology of citrus: I. Genomic determinants of evolution

**DOI:** 10.1002/tpg2.20104

**Published:** 2021-07-18

**Authors:** Daniel Gonzalez‐Ibeas, Victoria Ibanez, Estela Perez‐Roman, Carles Borredá, Javier Terol, Manuel Talon

**Affiliations:** ^1^ Instituto Valenciano de Investigaciones Agrarias (IVIA) Carretera Moncada CV‐315, Km 10 Valencia 46113 Spain

## Abstract

We performed genomic analyses on wild species of the genus *Citrus* to identify major determinants of evolution. The most notable effect occurred on the pathogen‐defense genes, as observed in many other plant genera. The gene space was also characterized by changes in gene families intimately related to relevant biochemical properties of citrus fruit, such as pectin modifying enzymes, *HDR* (4‐hydroxy‐3‐methylbut‐2‐enyl diphosphate reductase) genes, and O‐methyltransferases. Citrus fruits are highly abundant on pectins and secondary metabolites such as terpenoids and flavonoids, the targets of these families. Other gene types under positive selection, expanded through tandem duplications and retained as triplets from whole genome duplications, codified for purple acid phosphatases and MATE‐efflux proteins. Although speciation has not been especially rapid in the genus, analyses of selective pressure at the codon level revealed that the extant species evolved from the ancestral citrus radiation show signatures of pervasive adaptive evolution and is therefore potentially responsible for the vast phenotypic differences observed among current species.

## Core Ideas

- Major genomic determinants of the citrus biology targeted by evolution are determined.
- Evolution affected genes families regulating biochemical characteristics of citrus fruit.
- Pervasive adaptation played an important role during the citrus radiation.

**Abbreviations**
dN: nonsynonymous
dS: synonymous
BAM: Binary Alignment/Map
GO: Gene Ontology
HDR: 4‐hydroxy‐3‐methylbut‐2‐enyl diphosphate reductase
LRT: likelihood ratio test
OMT: O‐methyltransferase
SNP: single nucleotide polymorphism
VCF: Variant Call Format
WGD: whole genome duplications

## 1 INTRODUCTION

Evolution operates at a broad spectrum of genomic scale, from large chromosomal rearrangements to small changes affecting single nucleotides, and protein‐coding genes are still a major focus to understand how species adapt (Chen et al., 2018). From an applied research standpoint, evolutionary studies may help to identify specific gene families or members within each family that greatly influence the biology of the species and therefore constitute valuable resources for plant breeding programs.

Gene duplications, including both tandem and whole genome duplications (WGD), are of major relevance for evolution because they allow amplification and diversification of gene functions (Qiao et al., 2019). Duplicates retained after fractionation (i.e., return to the original ploidy) provide valuable insights on both gene relevance and speciation (Ren et al., 2018). Gene duplications play an important role not only during evolution but also in domestication. In maize (*Zea mays* L.), for example, thousand DNA segments, often including genic sequences, can be identified as presence/absence variations even between nearly identical genotypes (Springer et al., 2009), and structural variations were reported among citrus cultivars (Terol et al., 2015). Underlying the gene level, nucleotide changes and codon substitution models implemented within maximum likelihood and Bayesian frameworks have become the standard to infer the history of natural selection of a gene sequence at the molecular level (Anisimova & Kosiol, 2009). The power of these models can be increased by analyzing the effects among nucleotides (NSsites analysis) and lineages (branch analysis) to overcome the limitations of averaging substitution rates along the entire gene sequence (Anisimova & Kosiol, 2009). Several examples illustrate how such studies in genome‐wide scans may reveal new insights into episodic positive selection during speciation (Anisimova & Kosiol, 2009; Burri et al., 2010; Nielsen et al., 2005; Zhang, 2003) and identify critical amino acids targeted by evolution (Sawyer et al., 2005; Anisimova & Kosiol, 2009).

In regard to citrus, exploration of the evolutionary forces is timely because the genealogical relationships of ancestral citrus species and the parental relationships of cultivars were recently redefined (Wu et al., 2018). According to this annotation, ancestral citrus originated in the Himalayan foothills and diversified in a rapid radiation during the late Miocene to give rise to the current wild species; however, as of yet there is no evidence to indicate whether selective pressures played a major role. Although these ancestral relatives are pure accessions where no foreign introgressions were identified, edible cultivated citrus (oranges, grapefruits, and modern mandarins, including the *Citrus* reference *Citrus clementina*) are mostly admixtures of ancestral species of pummelo [*Citrus maxima* (Burr. f) Merr] and mandarin (*Citrus reticulata*), the latter of which are represented in our work by Tachibana (TBM) and Sun Chu Kat wild mandarins (SCM). Half of the genome of acidic cultivated citrus comes from ancestral citron (*Citrus medica* L.), whereas other nonedible cultivars are predominantly hybrids among these three ancestral species or other wild citrus. We analyzed the evolution of the shared gene space among selected wild pure accessions that were previously used for phylogenetic inference of citrus for selective pressure at the codon level to determine the extent to which adaptation has played a role in the recent citrus radiation, with a special focus on the three pivotal species (i.e., pummelo, mandarin, and citron) from which most current domesticated varieties derive. We combined analyses of presence/absence gene polymorphisms, duplications, and gene family overrepresentation in the whole set of genes to depict major determinants of evolution in citrus. It is currently accepted that domestication is a form of natural evolution and constitutes one of several driving forces that shape species biology (Meyer & Purugganan, 2013). Thus, we investigated the genomic signatures of both natural and artificial selection on wild and domesticated accessions, and, for simplicity, the results were divided in two papers. Here, we discuss the evolutionary determinants, while our companion paper focuses on the elucidation of domestication signatures (Gonzalez‐Ibeas et al., 2021).

**Core Ideas**

- Major genomic determinants of the citrus biology targeted by evolution are determined.
- Evolution affected genes families regulating biochemical characteristics of citrus fruit.
- Pervasive adaptation played an important role during the citrus radiation.

## 2 MATERIALS AND METHODS

### 2.1 Single nucleotide polymorphism identification

DNA HiSeq Illumina reads from whole genome sequencing projects were retrieved from repositories (Supplemental Table S7) and quality filtered with custom Python scripts (Phred score >30, read length 100 bp). Trimmed reads were mapped on the clementine haploid reference genome (v1.0, Phytozome; Goodstein et al., 2012) with bwa‐mem (v0.7.15‐r1140) (Li & Durbin, 2009), setting ‐r 1.2 option. Polymerase chain reaction duplicates were removed using Picard MarkDuplicates (v1.139) (http://broadinstitute.github.io/picard/) deployed in GATK (Van der Auwera et al., 2013). Only reads of mapping quality >10 and uniquely mapped on the genome were used for single nucleotide polymorphism (SNP) discovery. Single nucleotide polymorphisms were called with GATK HaplotypeCaller (v3.8‐1‐0) (Van der Auwera et al., 2013) with default parameters and with subsequent filtering as set in (Wu et al., 2018). Only bi‐allelic SNPs are used in this analysis. Variant Call Format (VCF) files were processed with the GATK tools for additional filtering, including read base quality score (≥30), quality by depth (≥2.0), Phred‐scaled *P*‐value using Fisher's exact test to detect strand bias (<60), read mapping quality (≥ 40), Z‐score From Wilcoxon rank sum test of Alt vs. Ref read mapping qualities (>−12.5), Z‐score from Wilcoxon rank sum test of Alt vs. Ref read position bias (>−8.0). VCFtools (v0.1.13) (Danecek et al., 2011) and custom Python scripts were also used to filter VCFs by position.

### 2.2 Selective pressure analysis and identification of the shared gene space

**FIGURE 1:**
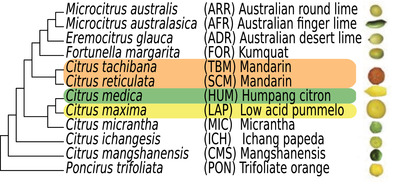
Phylogeny of *Citrus* (Wu et al., 2018) showing the 12 species under study. Species ancestral to most citrus cultivars include the citron (*Citrus medica* L., highlighted in green), pummelo [*Citrus maxima* (Burm. f.) Merr., highlight in yellow], and mandarins [*Citrus tachibana* (Makino) T. Tanaka and *Citrus reticulata* Blanco, highlighted in orange].

Selective pressure was analyzed in the 12 wild pure citrus species of the new established phylogeny (Figure 1). In addition to a well‐defined phylogeny, these analyses typically require and start by the identification of orthologs among the species to be analyzed. We took advantage of the high levels of collinearity and genome structure conservation among citrus (He et al., 2020), and we followed a mapping approach as a proxy of orthology. DNA HiSeq Illumina reads from whole genome sequencing projects deposited on repositories (Supplemental Table S7) were filtered (Phred quality base >30, length 100 bp) and mapped on the clementine reference genome (v1.0, Phytozome) (Goodstein et al., 2012) with bwa‐mem (v0.7.15‐r1140) (Li & Durbin, 2009) to generate Binary Alignment/Map (BAM) files, setting ‐r 1.2 option. Polymerase chain reaction duplicates were removed using Picard MarkDuplicates (v1.139) (http://broadinstitute.github.io/picard/) deployed in GATK (Van der Auwera et al., 2013). Reads flagged as properly paired were selected with Samtools (v1.9) (Li et al., 2009). Sequencing depth per nucleotide was also calculated with Samtools. Protein‐coding gene features were extracted from the GFF3 annotation files of the clementine reference genome (v1.0, Phytozome) (Goodstein et al., 2012). For each protein‐coding gene and for each sample, read coverage was estimated just for coding DNA sequences. Introns and untranslated regions (UTRs) were excluded. Next, percentage of coding nucleotides supported by at least four reads on average was used as the value of read coverage for each gene. Genes with 100% DNA read coverage (i.e., every nucleotide represented by DNA reads) for the 12 pure species simultaneously were defined as the “shared gene space” and used as orthologs for the analysis. The number of genes to be analyzed is probably an underestimation due to the stringent threshold, but this way we ensure that every single nucleotide of every gene is represented by sequencing data for proper data input for the next steps. The SNPs were identified from BAM files to generate the VCF files (see above). A consensus sequence of the genome for each sample was generated from VCF files with bcftools (Li et al., 2009). The nonsynonymous (dN)/synonymous (dS) rate ratios (ω = dN/dS) were calculated with codeml from the PAML package (v4.9) (Yang, 2007) for protein‐coding genes, taking the citrus phylogeny as reference. Overrepresented Gene Ontology (GO) categories in gene sets in relation to all GO terms from the whole set of annotated genes (GO annotation background) were identified with the Babelomics suit (v5.2.5) (Alonso et al., 2015).

### 2.3 Statistical models of selective pressure analysis with PAML

Models and evolutionary tests that were used included (a) the model M0 (one ratio), which assumes the same ω for all branches in the phylogeny and for all sites in the gene was utilized to estimate average omega values of genes. (b) The paired models M1a–M2a and M7–M8 were compared (M2a against M1a and M8 against M7, df = 2) to identify codons under positive selection when ω is allowed to vary among sites of the protein (NSsites models). Pair M1a–M2a was used as a replacement of the old M1–M2 models as suggested (Yang, 2007). The likelihood ratio test (LRT) was used to compare the log likelihoods of nested models (Anisimova & Kosiol, 2009) and statistically significant genes were identified according to the chi‐squared test, calculated with the chi2 tool from PAML. The Bayes Empirical Bayes procedure was used to identify the codons under positive selection when the LRT was significant and when the posterior probabilities for site classes were >0.95. The Naïve Empirical Bayes was not used in the calculations as suggested (Yang, 2007). (c) The two‐ratios branch model was used to identify genes under positive selection acting on particular lineages (defined as foreground lineages), because the omega ratio is allowed to vary among branches of the phylogeny. Lineages of interest (citron, pummelo, and mandarin) were flagged in the unrooted tree as foreground branches and the rest of species as background following PAML labeling rules of the phylogenetic tree in Newick format. The lineages were analyzed in independent runs. The analysis of mandarins was split into two additional runs: mandarins flagged as a single node (“node” mandarin) and mandarins flagged as separate branches (“clade” mandarin). (d) The branch‐site model A (so‐called alternative model) was used in the branch‐site test of positive selection affecting a few codons along particular lineages. Omega value was fixed to 1 in the null model and was estimated from data in the alternative model, and both were compared by LRT for significance. Citron, pummelo, and mandarins were set as foreground branches similarly to the branch model. The Bayes Empirical Bayes procedure was used to identify the codons under positive selection when the LRT was significant and when the posterior probabilities for site classes were >0.95. Finally, (e) the free‐ratios branch model (M1), which assumes independent ω values for each branch, has been successfully applied previously in citrus (Wang et al, 2017a), but it is considered very parameter‐rich by developers (Yang et al, 2007). Thus, in our work we used the two‐ratios branch model to get statistical significance at genome scale for particular branches of interest (i.e., citron, pummelo, and mandarin), while M1 model was only used to manually explore major evolutionary trends within each gene.

### 2.4 Analysis of novel lineage‐specific gene space

A reference genome assembly is available for pummelo (LAP; low acid pummelo) and citron (HUM; Humpang citron), but not for TBM and SCM. Thus, in order to keep consistency and avoid a bias against mandarins, a de novo genome sequence assembly was done for every sample. Unmapped reads on the clementine reference genome were extracted from the previously generated BAM files (see above, before any additional filtering) with samtools (v1.9) (Li et al., 2009) by using the flag 4. FASTQ files were recovered with the fastq option. Reads in FASTQ format were quality trimmed with Sickle (v1.33) (Joshi & Fass, 2011), setting read quality threshold to 30 and read length to 45 nt. Filtered reads were assembled with SPAdes (v3.11.1) (Bankevich et al., 2012) with default parameters. Only contigs of >1Kb in length were further analyzed. Contigs were blasted against the chloroplast (accession DQ864733.1) and mitochondrial (accession NC_037463.1) genome sequence from *C. sinensis*, and hits with >70% of length and >90% of nucleotide identity were considered of organelle source. Read depth coverage for each contig was calculated by mapping the input reads on assembled contigs with bowtie2 (Langmead & Salzberg, 2012) with default parameters. Contigs were annotated by blasting the DNA sequences against the nonredundant protein sequence database of GenBank (http://www.ncbi.nlm.nih.gov/genbank/) with tblastx (Altschul et al., 1990). Blast results in XML format were processed with the NCBIXML library of Biopython (v1.72) (Cock et al., 2009). Only hits with >90% of coverage, >50% sequence identity, and E‐value <1e‐05 were considered. Functional annotation of potential gene loci was carried out with Blast2GO (v5.2.4) (Conesa & Götz, 2008). Circos (v0.69‐8) (Krzywinski et al., 2009) was used for data visualization in circular layouts.

### 2.5 Gene duplication analysis

For whole genome duplication analysis, the clementine reference genome (v1.0, Phytozome) and the associated annotation files in GFF3 format were used as input for a self‐comparison analysis by aligning the genome against itself using LastZ (Harris, 2007). Two genes were used as the minimum number of aligned pairs for DAGchainer (Haas et al., 2004) during analysis of collinearity. Coding sequence divergence was measured via dS changes for homeologous protein coding gene pairs as calculated with codeml from the PAML package. Homeologs represented by three copies in identified syntelogs were used as WGD‐derived triplets. The same procedure was applied to identify homeologs in seven additional species within the Malvid clade by aligning their genomes with the clementine genome. The accessions included *Eucalyptus grandis* (v2.0, Phytozome), *Dimocarpus longan* (Lin et al., 2017), *Arabidopsis thaliana* (TAIR10, Phytozome), *Capsella rubella* (v1.0, Phytozome), *Carica papaya* (ASGPBv0.4, Phytozome), *Gossypium raimondii* (v2.0 Phytozome), *Theobroma cacao* (v1, https://cocoa‐genome‐hub.southgreen.fr). Overrepresented GO categories in gene sets were identified with the Babelomics suite (v5.2.5) (Alonso et al., 2015). Syntelog visualization and plotting was done with the GenomeDiagram Python library (Pritchard et al., 2006). Gene duplicates in tandem were retrieved from the raw output of the CoGe package based on gene annotations in the reference clementine genome. Gene presence/absence polymorphisms were estimated in pure accessions (citrus phylogeny), hybrids, and admixtures according to their mapping coverage in the different samples (the same method used to identify the “shared gene space”, see above, but applying different read coverage cut‐offs). Genes with read coverage of >90% in a sample were considered as “present”, with <10% were considered as “absent,” and ranging from 10 to 90% were flagged as “diverged” or containing structural variants. Gene coverage data were plotted as a heat map (samples vs. genes) with Python. For identification of protein domains overrepresented in citrus, Pfam domain data sets were downloaded from PLAZA (dicots v04) (Van Bel et al., 2018) for organisms listed on Supplemental Table S4. Phylogenetic analysis of protein sequences was performed with MUSCLE (v3.8.31) (Edgar, 2004) for protein sequence alignment, RaxML (v8.2.11+dfsg‐1) (Stamatakis, 2014) for tree generation ,and FigTree (v1.4.4) (http://tree.bio.ed.ac.uk/software/figtree/) for tree visualization.

## 3 RESULTS AND DISCUSSION

### 3.1 Selective pressure at the codon level

DNA reads from 12 pure citrus species were mapped to the clementine genome (Wu et al., 2014), identifying an average of 857,214 SNPs per sample (Figure 1). There was high variability among accessions, ranging from 398,359 SNPs in *C. tachibana* (Makino) T. Tanaka to 1,159,792 SNPs in *Poncirus trifoliata* (L.) Raf. Coding DNA sequences had the lowest percentage of polymorphisms, compared to UTRs and introns. We identified a subset of 13,199 genes (54% of 24,533 annotated loci) with full read coverage in all species, defined from now on as the shared gene space, which was further analyzed for selective pressure at the codon level. Nucleotide substitution rates of dN and dS sites were explored with PAML from three perspectives: evolution across the whole tree, per branches and per nucleotide sites. Omega values of all genes for the whole citrus tree showed a typical distribution with most genes subject to purifying constraints (ω < 1) (Supplemental Table S1.0). Selective pressure per branches was analyzed for the three parental species of domesticated citrus, namely mandarin, citron, and pummelo, by means of the free‐ratio (Supplemental Table S1.1), branch (Supplemental Table S1.2), and branch‐site (Supplemental Table S1.3) PAML analysis to identify episodic positive selection. At most, only a few tens of genes were identified under positive selection through the branch analysis and no significant genes were retrieved by the branch site test (Supplemental Table S1.2). In contrast, 9,676 genes showed significant evidence of codons evolving under pervasive selection (NSsites analysis), where 4,377 of these genes had identifiable evolving codons (Supplemental Table S1.4), ranging from 1 to 55 codons/gene (4 on average), affecting 24,210 amino acids.

**FIGURE 2:**
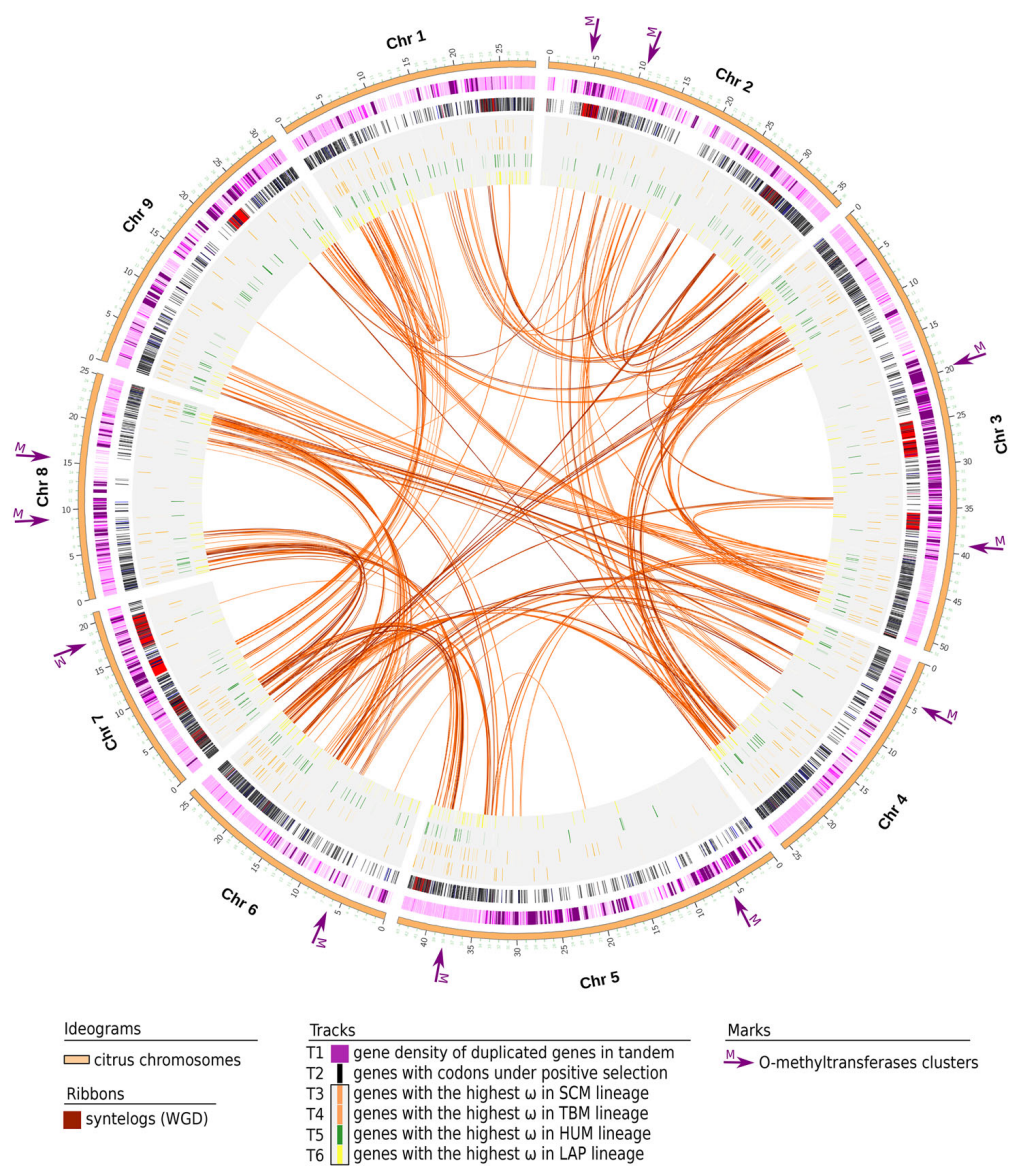
Circos diagram of the clementine (*Citrus clementina*) genome, the reference genome for citrus (Wu et al., 2014). Each ideogram represents a chromosome. Track 1 (T1) represents density of gene duplications in tandem. The more intense the purple color, the higher the number of genes contained. Clusters of O‐methyltransferases (OMTs) are highlighted with purple arrows. Track 2 (T2) represents genes where at least one codon has been identified under selective pressure by NSsites–PAML analysis. Red areas contain genes that held the highest number of evolving codons. Tracks 3–6 represent genes where omega (ω) value, calculated by the free‐ratios PAML analysis, was substantially greater in one lineage relative to other lineages. Ribbons connect duplicated areas (syntelogs) derived from the paleohexaploidy event shared by eudicots

Genes subject to any type of selective force were evenly distributed along the genome, except for being nearly absent in centromeric areas. Some minor biases were observed, for example, lower densities of positively selected genes at the start of chromosomes 5 and 6. However, as these gene clusters were absent in some species and therefore outside of the shared gene space, they were excluded from the analysis (Figure 2). Genes were ranked according to the number of codons evolving under selection or their associated *P*‐value in order to identify overrepresented GO categories by the logistic model of the Babelomics suite (Supplemental Table S2). Genes involved in host defense against pathogens (e.g., *NBS*‐*LRR* genes, including both Toll/Interleukin‐1 receptor resistenance protein and coiled‐coil domains) comprised one of the main categories identified. Protein phosphorylation and cellular signaling (generally kinases) GO categories were also identified, as well as those related to carbohydrate metabolism, including starch and sucrose metabolism, and to the regulation of cell wall biosynthesis. This last category was represented by pectin modifying enzymes (pectin lyases, pectin methylesterases, and their cognate inhibitors) and genes implicated in cellulose biosynthesis. Ion transmembrane transport (GO:0034220), including glutamate receptors and cyclic nucleotide‐gated ion channels, was also an overrepresented category. On the contrary, GO terms related to basic cellular processes were mostly represented in the set of genes with a low proportion of codons under selection, such as translation (GO:0006412), ribosome biogenesis (GO:0042254), electron transport (GO:0006118), or regulation of translational initiation (GO:0006446). Other genes under positive selection potentially related to the citrus biology were two genes coding for citrate lyases and four genes of isocitrate dehydrogenases. Genes under selective pressure that are related to other findings reported in the next sections are, for instance, MATE‐efflux transporters and purple acid phosphatases.

### 3.2 WGD

Unlike many other angiosperms, no recent WGD has been reported in citrus; however, remnants of the ancient hexaploidy (aka gamma) event shared by all eudicots can be tracked. A self‐genome comparison of sequence similarity and collinearity of the clementine genome revealed that 3,433 genes were organized in 246 syntelogs, ranging from 51 Kb to 1.8 Mb in length (358 Kb on average). Out of these, 612 genes were located in regions syntenic to two other intragenomic regions, that is, they were structured in 204 triplets that grouped in 68 syntelogs. The genome organization of this set of syntelogs, which was used for downstream analysis, was highly shuffled along all chromosomes (Figure 2). Seventy nine percent of these genes were supported by expression data (Terol et al., 2016), and 73% showed 100% read coverage (99.6% on average), revealing extremely high gene retention and sequence conservation (Supplemental Figure S1b1), in agreement with other works (Yuan et al., 2019). We further extended the analysis by the same procedure to seven additional Malvids outside the genus *Citrus* to analyze citrus speciation in phylogenetically close relatives. In contrast to citrus, the number of shared genes was highly variable among these Malvid species, ranging from 8 in *Arabidopsis* to 229 in cocoa (Supplemental Figure S1a2).

**FIGURE 3:**
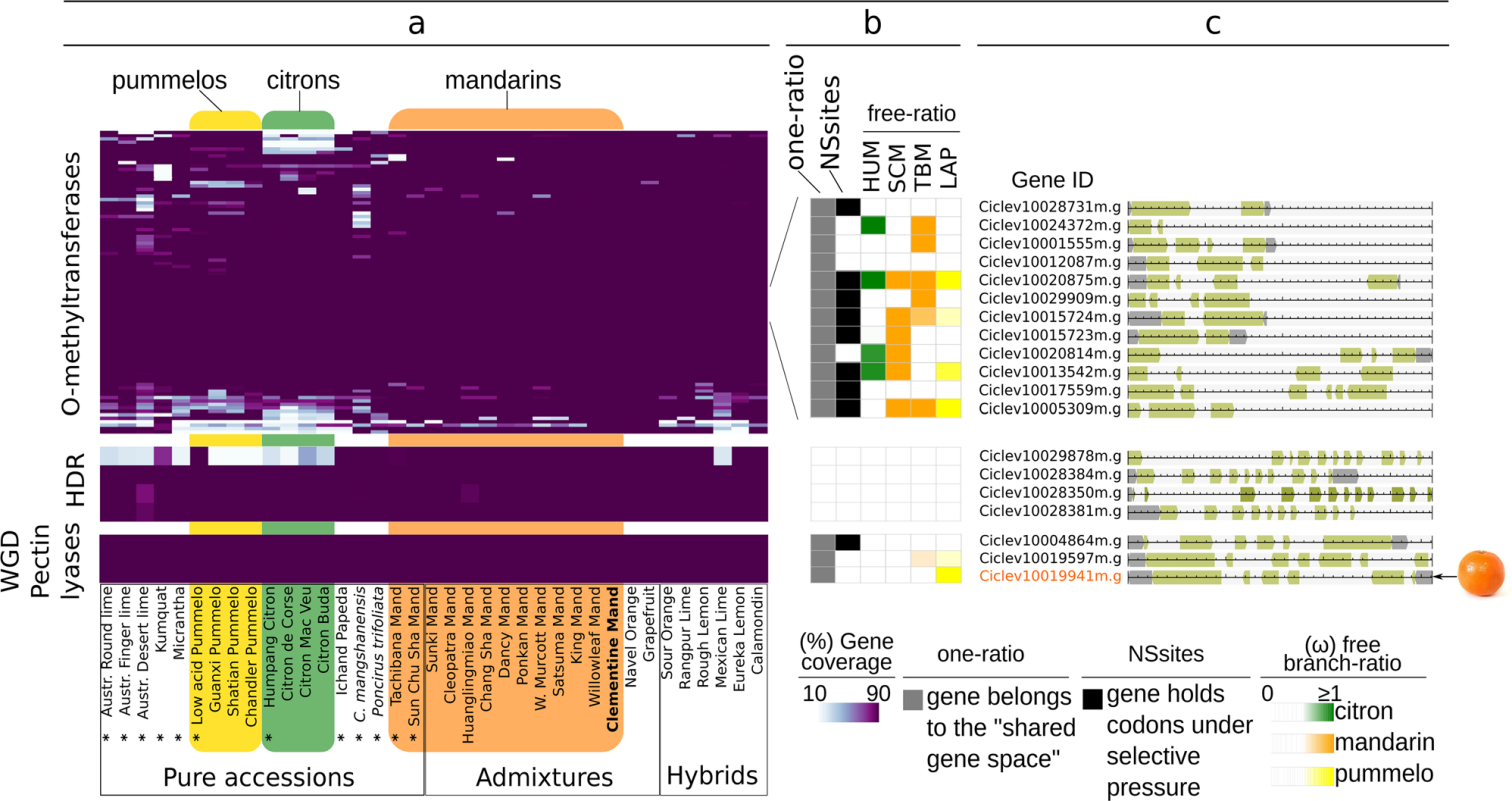
(a) Presence/absence polymorphism of O‐methyltransferases, *HDR* genes, and whole genome duplication (WGD)‐derived pectin lyases (*y*‐axis, one gene per row) in different citrus accessions (*x*‐axis), represented by a color scale of DNA read mapping coverage (color = gene present; white = gene absent). Genomes of pure citrus species used in the phylogenetic tree presented in Figure 1 are highlighted with an asterisk, whereas the clementine (*Citrus clementina*) reference is highlighted in bold. (b) Codon selective pressure summary: gene belongs to the shared gene space (grey, 1st column); gene with codons identified under selective pressure by the NSsites codeml analysis (black, 2nd column); branch–omega value calculated by the free‐ratio codeml analysis (columns 3–6) in Humpang citron (HUM, green), Tachibana (TBM, orange), Sun Chu Sha (SCM, orange), and low acid pummelo (LAP, yellow). Gene *Ciclev10019941m.g* (flagged with a mandarin image) showed a markedly higher free‐ratios‐omega value in pummelo and was identified as potentially involved in fruit size determination as a part of the analysis of single nucleotide polymorphism (SNP) data and pummelo introgression (companion publication, Gonzalez‐Ibeas et al., 2021). (c) Representation of the exon/intron structure: green = exon, grey = untranslated regions (UTR), empty = intron. Gene length is scaled to the longest one.

Gene retention from the paleohexaploidy event and high conservation among citrus species implies relevance, but some of these genes are more easily related to the biological features of citrus due to their molecular function or the biological process that they are involved in, such as purple acid phosphatases, pectin methylesterase inhibitors, phenylalanine ammonia lyases, MATE efflux proteins, and squalene epoxidases (Supplemental Table S3, see Discussion). A triplet syntelog of pectin lyase genes is fully conserved in all pure citrus species but evolved differentially at the level of selective pressure in pummelo (Figure 3b) and potentially related to citrus fruit size regulation (Gonzalez‐Ibeas et al., 2021).

### 3.3 Gene duplication, gain and loss in citrus

Tandem gene duplication deeply affected the clementine genome as expected, because a total of 13,357 genes (nearly 50%) were grouped in 3,250 clusters (Supplemental Table S4). Clusters included 2 to 52 genes (4 genes on average) and ranged from 1 to 134 (3 clusters on average) per protein domain (Figure 2, Track T1). Main categories included kinases, leucine‐reach repeats, pathogen‐resistance genes, cytochrome P450, pentatricopeptide repeats, and methyltransferases. In general, the number of clusters per family correlated positively with gene family size, pointing to tandem duplication as the main mechanism of expansion. Similar to the selective pressure analysis (see above), pathogen‐resistance genes were greatly affected by tandem expansion and presence/absence polymorphism among accessions, with the xerophyte *Eremocitrus glauca* representing the most extreme case (Supplemental Figure S2).

Predicted citrus protein domains were compared with 51 noncitrus organisms to identify overrepresented domains, which would have a higher likelihood of being citrus‐specific determinants. A complete list of results is provided in Supplemental Table S4. The largest expansion occurred within the O‐methyltransferases (OMTs) of Family 2 (domain PF00891; Figure 3), which expanded largely in tandem (80% of members in 20 clusters; Figure 2, purple arrows). Functional annotation characterized half of these proteins as caffeic acid methyltransferases, and the other half as methylases of small phenolic acids (e.g., orcinol and eugenol) and more complex flavonoids (e.g., quercetin) (see Supplemental Text S1). Another interesting expansion was that of the *HDR* (4‐hydroxy‐3‐methylbut‐2‐enyl diphosphate reductase; also called LytB) gene, involved in terpenoid biosynthesis, that exhibits up to four copies in citrus genomes, the largest expansion in the set of plants analyzed here, because most of them only harbor one copy (Supplemental Table S4). Within the Malvid clade, the expansion appears to be relatively recent and specific to citrus, as the four citrus genes grouped by species instead of following orthologous relationships (Supplemental Figure S3). Indeed, this gene seems to have undergone recurrent expansions along evolution in a few plant species with high terpenoid content. Thus, it was expanded to three copies in carrot (*Daucus carota* L.) and pepper (*Capsicum annuum* L.) (Supplemental Table S4), two species characterized by high carotenoid content, similar to citrus. The genome of cotton (Gossypium L.) (a species that shows elevated levels of mono‐ and sesquiterpenoids) also harbors three copies of this gene. It is worth noting that *Arabidopsis* has only one copy after two rounds of lineage‐specific WGD, which further suggests that this gene expanded in several nonrelated species likely on the basis of functional requirements.

Furthermore, HDR copies in citrus were located in tandem to form a cluster (Supplemental Figure S3B), in contrast to other species examined (*Brassica rapa* L., and carrot had two and three copies, respectively, located on two different chromosomes), suggesting that those expansions occurred by different mechanisms. Presence/absence polymorphisms in citrus showed that one of the *HDR* genes (*Ciclev10029878m.g*) was not present in citrons, pummelos (except low‐acid pummelo), Australian lime (*Microcitrus* sp.), or Micrantha (*Citrus micrantha*, Wester), suggesting copy number variation within the stable genome architecture of citrus. The proper exon/intron structure of the genes was not suggestive of pseudogenization (Figure 3C), and all four genes are expressed in mandarins and oranges (unpublished data, 2020), although our data indicate one single gene, *Ciclev10028384m.g*, dominated the expression profile.

Albeit not expanded in citrus, some gene families shared both WGD and tandem duplications, for example, MATE‐efflux proteins, which are involved in membrane transport of several compounds, including anthocyanidins and flavonoids (Santos et al., 2017). The 68 citrus gene products carrying a PF01554 domain typical of MATE proteins were clustered with sequences of 35 experimentally well‐characterized MATE proteins (Liu et al., 2016a), forming a phylogenetic tree displaying the occurrence of four well separate MATE groups named C1–C4 (Supplemental Figure S4). Family C1 (anthocyanin and flavonoid transport), which is expanded in citrus relative to the other three families (29 vs. 21, 9, or 9 members), was also characterized by differential gene presence/absence polymorphism in pure citrus species, as well as in mandarins and derived mandarin admixtures. Interestingly, the C1 family contained a subcluster with 17 members, suggesting citrus specificity (Supplemental Figure S4, C1.1 grey box).

DNA reads from the pure citron (HUM), pummelo (LAP), and mandarin (TBM and SCM) genomes that did not map to the clementine reference sequence were assembled and annotated in order to identify potential lineage‐specific genes within the citrus clade. Over 11 million reads were assembled onto 2,627 scaffolds ranging from 2,500 to 41,877 bp in length with an N50 of 6 Kb on average (Supplemental Table S5). Pathogen resistance genes were once again identified in the novel gene set (17 genes), as were transposable elements (34 loci). In particular, transposons TNT 1–94 were especially abundant (Supplemental Table S6). More interestingly, genes involved in secondary metabolism were also represented, such as an isoflavone 2′‐hydroxylase, a coumarin 8‐geranyltransferase, two geraniol 8‐hydroxylases, four cytochromes P450, one acyl carrier protein (fatty acid biosynthesis), one 3‐ketoacyl‐CoA thiolase (fatty acid oxidation), one (R)‐limonene synthase, and one (R)‐mandelonitrile lyase (cyanogenic glycosides metabolism). Surprisingly only four transcription factors were found.

## 4 DISCUSSION

In this work, we present a study of the genomic determinants of evolution of the citrus gene space. The most notable effect occurred in the pathogen‐defense genes, as observed in many other genera (Zhang et al., 2019). Although this phenomenon is general in plants, our study also identified gene determinants more specific to citrus. Thus, selective pressure also affected genes of enzymes that modify pectins, which are major components of the cell wall and their changes are associated with cellular adhesion, wall plasticity and growth, and the control of pH and ionic contents (Braidwood et al., 2014; Peaucelle et al., 2008; Pelloux et al., 2007). In citrus, pectins are highly abundant in the fruit peel and specially in the white albedo, the fruit mesocarp. Because the importance of pectin biosynthesis and degradation during citrus fruit growth and ripening is broadly documented (Collins et al., 2019; Guillon et al., 2017), it is reasonable to speculate that modification of these structural components could contribute to the high morphological diversity of citrus. The expansion of *HDR* genes is the largest one reported in plants and presumably linked to the high array of terpenoids in citrus. This enzyme catalyzes the last step of the nonmevalonate (2‐C‐methyl‐D‐erythritol 4‐phosphate/1‐deoxy‐D‐xylulose 5‐phosphate) pathway, which takes place in the chloroplast and leads to the synthesis of isoprenoid precursors. Thus, the HDR expansion appears to be related to the high production of more than 200 different citrus compounds derived from prenyl diphosphate precursors that are synthesized in citrus (González‐Mas et al., 2019; Ladanyia & Ladaniya, 2010), mostly monoterpenes, sesquiterpenes, and their oxygenated derivatives. Examples of these compounds include d‐limonene, which accounts for 80–95% of all citrus oils, and valencene. Furthermore, citrus contain a great deal of triterpenes such as those belonging to the group of limonoids (e.g., limonin), responsible for the bitter taste of the juice, and a complex set of carotenoids (carotenes and xanthophylls) that provides the external and internal fruit coloration (Lado et al., 2019). Alternatively, to a net increase in biosynthesis capabilities, the citrus *HDR* expansion may rather have led to a sophistication in the mevalonate pathway to fulfill the complex array of isoprenoid derivatives.

The expansion of OMTs, as related to the other plant species, appears to be linked to citrus aromas and polymethoxyflavone modifications. This amplification in citrus has been reported before by Liu et al. (2016b), who associated some members with the occurrence of isoeugenol and lignin synthesis in *Citrus sinensis*. Citrus fruit aroma consists mainly of mono and sesquiterpenes, although nonterpenoids still represent a small but significant percentage. Eugenol, for example, a “sweet/honey” aroma‐impact compound based on olfactometry studies (Lin & Rouseff, 2001), which has insecticide properties (Huang et al., 2002), is methylated by OMTs as part of aroma production in other species (Wu et al., 2003). In addition to small phenolics, OMTs also participate in the modification of polymethoxyflavones, a type of highly methylated flavonoids identified in the peel of sweet orange and wild mandarins (Lei et al., 2017). In fact, citrus fruit contain approximately 200 flavonoids and no less than 50 of them are methylated or polymethoxylated compounds (Wang et al., 2017b). Similarly, a large expansion of OMTs has also been reported in *Eucalyptus*, where polymethoxyflavones are abundant (Goodger et al., 2016). Moreover, OMTs have been experimentally confirmed to be involved in the biogenesis of these compounds in sweet basil (*Ocimum basilicum* L.) (Berim et al., 2012).

Other gene types under selection pressure, which expanded through tandem duplications and were retained as triplets from the gamma WGD event, code for MATE‐efflux proteins or purple acid phosphatases. MATE‐efflux proteins are a relevant set of the multidrug efflux transporters that transfer a broad range of organic substrates including anthocyanidins and flavonoids (Santos et al., 2017). The expansion of particular MATE families may be related to the wide array of flavonoids detected in citrus. Acid phosphatases are in principle involved in phosphate metabolism (Wang et al., 2014), although other roles in the acidic vacuoles of the citrus fruit cell, such as those proposed for animal acid endosomes, or cell wall remodeling (Kaida et al., 2010) cannot be discarded. Other expanded gene families reported in Supplemental Table S4 are more difficult to link to biological traits of citrus a priori, but they highlight elements to drive further research. For instance, the checkpoint protein Hus1/Mec3 may relate to the high and potentially active transposon repertoire of citrus, an activity that has been recently involved in citrus speciation (Borredá et al., 2019).

The WGD triplets analyzed here have relevant evolutionary implications. Gene loss and genome rearrangements during diploidization often accompany speciation (Panchy et al., 2016). Their conservation in citrus, even comparing with phylogenetically close species, indicates that diversification within the genus has involved rather small genomic changes, and relatively low rates of speciation, despite the huge phenotypic diversity observed. This avenue is also supported by the lack of an early WGD, the low number of lineage‐specific genes detected among the ancestral species analyzed (citron, pummelo, SCM, and TBM), and the high rates of gene flow described among all citrus (Gonzalez‐Ibeas et al., 2021), which denote incomplete reproductive isolation. This speculation agrees that some adaptive radiations have little or no effect on speciation rate (Givnish, 2015). Moreover, members of adaptive radiations often display higher rates of apparent positive selection than in nonradiating groups (Nevado et al., 2019). Citrus presents an example of adaptive radiation with a low rate of speciation: 39% of citrus genes appear to involve adaptive evolution by NSites analysis, a high proportion and comparable to other genera recognized as having undergone adaptive radiation on islands or mountaintops (Nevado et al., 2019). The much lower number of genes identified as being under selection in particular lineages within *Citrus* argues that diversifying evolution has been pervasive, at least for the three species that gave rise to the domesticated cultivars.

In conclusion, this work shows that the attributes characterizing the current citrus fruit, namely, the abundance of pectin and secondary metabolites such as terpenoids and flavonoids, are the result of large gene family expansions that evolved from the ancestral citrus genome. The evolution of pectin‐modifying enzymes, *HDR* genes, OMTs, acid phosphatases, and MATE‐efflux transporters, for instance, is significantly associated with selection pressure, tandem duplications, or triplet retention from the gamma event. Our results also reveal that extant species evolved from the ancestral citrus radiation show signatures of pervasive adaptive evolution, although speciation has not been especially rapid in citrus. This observation is particularly interesting as citrus diversification involved rather small genomic changes, despite the vast phenotypic differences observed in the genus.

## AUTHOR CONTRIBUTIONS

Daniel Gonzalez‐Ibeas: Conceptualization; Formal analysis; Investigation; Methodology; Software; Visualization; Writing‐original draft; Writing‐review & editing. Victoria Ibanez: Validation. Estela Perez‐Roman: Validation. Carles Borredá: Validation. Javier Terol: Funding acquisition; Project administration. Manuel Talon: Conceptualization; Funding acquisition; Investigation; Project administration; Supervision; Writing‐original draft; Writing‐review & editing.

## CONFLICT OF INTEREST

The authors declare no conflict of interest.

## Supporting information

Supplementary figures.

Table S1: Selective pressure analysis at the codon level results. Tables S1.0‐S1.4 are in several sheets of the same file.

Table S4: Comparison of the protein domain abundance in 51 organisms

Table S6: Lineage‐specific gene identification

Table S7: Sample description and GenBank accessions

## References

[tpg220104-bib-0001] Alonso, R. , Salavert, F. , Garcia‐Garcia, F. , Carbonell‐Caballero, J. , Bleda, M. , Garcia‐Alonso, L. , Sanchis‐Juan, A. , Perez‐Gil, D. , Marin‐Garcia, P. , Sanchez, R. , Cubuk, C. , Hidalgo, M. R. , Amadoz, A. , Hernansaiz‐Ballesteros, R. D. , Alemán, A. , Tarraga, J. , Montaner, D. , Medina, I. , & Dopazo, J. (2015). Babelomics 5.0: Functional interpretation for new generations of genomic data. Nucleic Acids Research, 43, W117–W121. 10.1093/nar/gkv384 25897133 PMC4489263

[tpg220104-bib-0002] Altschul, S. F. , Gish, W. , Miller, W. , Myers, E. W. , & Lipman, D. J. (1990). Basic local alignment search tool. Journal of Molecular Biology, 215, 403–410. 10.1016/S0022-2836(05)80360-2 2231712

[tpg220104-bib-0003] Anisimova, M. , & Kosiol, C. (2009). Investigating protein‐coding sequence evolution with probabilistic codon substitution models. Molecular Biology and Evolution, 26, 255–271. 10.1093/molbev/msn232 18922761

[tpg220104-bib-0004] Bankevich, A. , Nurk, S. , Antipov, D. , Gurevich, A. A. , Dvorkin, M. , Kulikov, A. S. , Lesin, V. M. , Nikolenko, S. I. , Pham, S. , Prjibelski, A. D. , Pyshkin, A. V. , Sirotkin, A. V. , Vyahhi, N. , Tesler, G. , Alekseyev, M. A. , & Pevzner, P. A. (2012). SPAdes: A new genome assembly algorithm and its applications to single‐cell sequencing. Journal of Computational Biology, 19, 455–477. 10.1089/cmb.2012.0021 22506599 PMC3342519

[tpg220104-bib-0005] Berim, A. , Hyatt, D. C. , & Gang, D. R. (2012). A set of regioselective O‐methyltransferases gives rise to the complex pattern of methoxylated flavones in sweet basil. Plant Physiology, 160, 1052–1069. 10.1104/pp.112.204164 22923679 PMC3461529

[tpg220104-bib-0006] Borredá, C. , Pérez‐Román, E. , Ibanez, V. , Terol, J. , & Talon, M. (2019). Reprogramming of retrotransposon activity during speciation of the genus *Citrus* . Genome Biology and Evolution, 11, 3478–3495. 10.1093/gbe/evz246 31710678 PMC7145672

[tpg220104-bib-0007] Braidwood, L. , Breuer, C. , & Sugimoto, K. (2014). My body is a cage: Mechanisms and modulation of plant cell growth. New Phytologist, 201, 388–402. 10.1111/nph.12473 24033322

[tpg220104-bib-0008] Burri, R. , Salamin, N. , Studer, R. A. , Roulin, A. , & Fumagalli, L. (2010). Adaptive divergence of ancient gene duplicates in the avian MHC Class II β. Molecular Biology and Evolution, 27, 2360–2374. 10.1093/molbev/msq120 20463048

[tpg220104-bib-0009] Chen, F. , Dong, W. , Zhang, J. , Guo, X. , Chen, J. , Wang, Z. , Lin, Z. , Tang, H. , & Zhang, L. (2018). The sequenced angiosperm genomes and genome databases. Frontiers in Plant Science, 9, 418. 10.3389/fpls.2018.00418 29706973 PMC5909171

[tpg220104-bib-0010] Cock, P. J. A. , Antao, T. , Chang, J. T. , Chapman, B. A. , Cox, C. J. , Dalke, A. , Friedberg, I. , Hamelryck, T. , Kauff, F. , Wilczynski, B. , & De Hoon, M. J. L. (2009). Biopython: Freely available Python tools for computational molecular biology and bioinformatics. Bioinformatics, 25, 1422–1423. 10.1093/bioinformatics/btp163 19304878 PMC2682512

[tpg220104-bib-0011] Collins, P. P. , O'donoghue, E. M. , Rebstock, R. , Tiffin, H. R. , Sutherland, P. W. , Schröder, R. , Mcatee, P. A. , Prakash, R. , Ireland, H. S. , Johnston, J. W. , Atkinson, R. G. , Schaffer, R. J. , Hallett, I. C. , & Brummell, D. A. (2019). Cell type‐specific gene expression underpins remodelling of cell wall pectin in exocarp and cortex during apple fruit development. Journal of Experimental Botany, 70, 6085–6099. 10.1093/jxb/erz370 31408160

[tpg220104-bib-0012] Conesa, A. , & Götz, S. (2008). Blast2GO: A comprehensive suite for functional analysis in plant genomics. International Journal of Plant Genomics, 2008, Article 619832. 10.1155/2008/619832 PMC2375974 18483572

[tpg220104-bib-0013] Danecek, P. , Auton, A. , Abecasis, G. , Albers, C. A. , Banks, E. , Depristo, M. A. , Handsaker, R. E. , Lunter, G. , Marth, G. T. , Sherry, S. T. , Mcvean, G. , & Durbin, R. (2011). The Variant Call Format and VCFtools. Bioinformatics, 27, 2156–2158. 10.1093/bioinformatics/btr330 21653522 PMC3137218

[tpg220104-bib-0014] Edgar, R. C. (2004). MUSCLE: Multiple sequence alignment with high accuracy and high throughput. Nucleic Acids Research, 32, 1792–1797. 10.1093/nar/gkh340 15034147 PMC390337

[tpg220104-bib-0015] Givnish, T. J. (2015). Adaptive radiation versus “radiation” and “explosive diversification”: Why conceptual distinctions are fundamental to understanding evolution. The New Phytologist, 207, 297–303. 10.1111/nph.13482 26032979

[tpg220104-bib-0016] Gonzalez‐Ibeas, D. , Ibanez, V. , Perez‐Roman, E. , Borredá, C. , Terol, J. , & Talon, M. (2021). Shaping the biology of citrus: II. Genomic determinants of domestication. The Plant Genome. 10.1002/tpg2.20111 PMC12806996 34275210

[tpg220104-bib-0017] González‐Mas, M. C. , Rambla, J. L. , López‐Gresa, M. P. , Blázquez, M. A. , & Granell, A. (2019). Volatile compounds in citrus essential oils: A comprehensive review. Frontiers in Plant Science, 10, 12. 10.3389/fpls.2019.00012 30804951 PMC6370709

[tpg220104-bib-0018] Goodger, J. Q. D. , Seneratne, S. L. , Nicolle, D. , & Woodrow, I. E. (2016). Foliar essential oil glands of *Eucalyptus* subgenus *Eucalyptus* (Myrtaceae) are a rich source of flavonoids and related non‐volatile constituents. PLOS ONE, 11, e0151432. 10.1371/journal.pone.0151432 26977933 PMC4792381

[tpg220104-bib-0019] Goodstein, D. M. , Shu, S. , Howson, R. , Neupane, R. , Hayes, R. D. , Fazo, J. , Mitros, T. , Dirks, W. , Hellsten, U. , Putnam, N. . , & Rokhsar, D. S. (2012). Phytozome: A comparative platform for green plant genomics. Nucleic Acids Research, 40, D1178–D1186. 10.1093/nar/gkr944 22110026 PMC3245001

[tpg220104-bib-0020] Guillon, F. , Moïse, A. , Quemener, B. , Bouchet, B. , Devaux, M. ‐. F. , Alvarado, C. , & Lahaye, M. (2017). Remodeling of pectin and hemicelluloses in tomato pericarp during fruit growth. Plant Science: An International Journal of Experimental Plant Biology, 257, 48–62. 10.1016/j.plantsci.2017.01.008 28224918

[tpg220104-bib-0021] Haas, B. J. , Delcher, A. L. , Wortman, J. R. , & Salzberg, S. L. (2004). DAGchainer: A tool for mining segmental genome duplications and synteny. Bioinformatics (Oxford, England), 20, 3643–3646. 10.1093/bioinformatics/bth397 15247098

[tpg220104-bib-0022] Harris, R. S. (2007). Improved pairwise alignment of genomic DNA [PhD Thesis]. Pennsylvania State University.

[tpg220104-bib-0023] He, L.i , Zhao, H. , He, J. , Yang, Z. , Guan, B. , Chen, K. , Hong, Q. , Wang, J. , Liu, J. , & Jiang, J. (2020). Extraordinarily conserved chromosomal synteny of Citrus species revealed by chromosome‐specific painting. The Plant Journal: For Cell and Molecular Biology, 103, 225–235. 10.1111/tpj.14894 32578280

[tpg220104-bib-0024] Huang, Y. , Ho, S. ‐. H. , Lee, H. ‐. C. , & Yap, Y. ‐. L. (2002). Insecticidal properties of eugenol, isoeugenol and methyleugenol and their effects on nutrition of *Sitophilus zeamais* Motsch. (Coleoptera: Curculionidae) and *Tribolium castaneum* (Herbst) (Coleoptera: Tenebrionidae). Journal of Stored Products Research, 38, 403–412. 10.1016/S0022-474X(01)00042-X

[tpg220104-bib-0025] Joshi, N. A. , & Fass, J. N. (2011). *Sickle: A sliding‐window, adaptive, quality‐based trimming tool for FastQ Files* (Version 1.33) [Software]. https://github.com/najoshi/sickle

[tpg220104-bib-0026] Kaida, R. , Serada, S. , Norioka, N. , Norioka, S. , Neumetzler, L. , Pauly, M. , Sampedro, J. , Zarra, I. , Hayashi, T. , & Kaneko, T. S. (2010). Potential role for purple acid phosphatase in the dephosphorylation of wall proteins in tobacco cells. Plant Physiology, 153, 603–610. 10.1104/pp.110.154138 20357138 PMC2879787

[tpg220104-bib-0027] Krzywinski, M. , Schein, J. , Birol, I. , Connors, J. , Gascoyne, R. , Horsman, D. , Jones, S. J. , & Marra, M. A. (2009). Circos: An information aesthetic for comparative genomics. Genome Research, 19, 1639–1645. 10.1101/gr.092759.109 19541911 PMC2752132

[tpg220104-bib-0028] Ladanyia, M. , & Ladaniya, M. (2010). Citrus fruit: Biology, technology and evaluation. Academic Press.

[tpg220104-bib-0029] Lado, J. , Alós, E. , Manzi, M. , Cronje, P. J. R. , Gómez‐Cadenas, A. , Rodrigo, M. J. , & Zacarías, L. (2019). Light Regulation of Carotenoid Biosynthesis in the Peel of Mandarin and Sweet Orange Fruits. Frontiers in Plant Science, 10. 10.3389/fpls.2019.01288 PMC6803510 31681382

[tpg220104-bib-0030] Langmead, B. , & Salzberg, S. L. (2012). Fast gapped‐read alignment with Bowtie 2. Nature Methods, 9, 357–359. 10.1038/nmeth.1923 22388286 PMC3322381

[tpg220104-bib-0031] Lei, J. , Xue, Y. , Liu, Y.i‐M. , & Liao, X. (2017). Characterization of major metabolites of polymethoxylated flavonoids in Pericarpium Citri Reticulatae using liver microsomes immobilized on magnetic nanoparticles coupled with UPLC/MS–MS. Chemistry Central Journal, 11, Article 13. 10.1186/s13065-017-0237-9 PMC5293709 28224016

[tpg220104-bib-0032] Li, H. , & Durbin, R. (2009). Fast and accurate short read alignment with Burrows–Wheeler transform. Bioinformatics, 25, 1754–1760. 10.1093/bioinformatics/btp324 19451168 PMC2705234

[tpg220104-bib-0033] Li, H. , Handsaker, B. , Wysoker, A. , Fennell, T. , Ruan, J. , Homer, N. , Marth, G. , Abecasis, G. , & Durbin, R. , & 1000 Genome Project Data Processing Subgroup . (2009). The sequence alignment/map format and SAMtools. Bioinformatics, 25, 2078–2079. 10.1093/bioinformatics/btp352 19505943 PMC2723002

[tpg220104-bib-0034] Lin, J. , & Rouseff, R. L. (2001). Characterization of aroma‐impact compounds in cold‐pressed grapefruit oil using time–intensity GC–olfactometry and GC–MS. Flavour and Fragrance Journal, 16, 457–463. 10.1002/ffj.1041

[tpg220104-bib-0035] Lin, Y. , Min, J. , Lai, R. , Wu, Z. , Chen, Y. , Yu, L. , Cheng, C. , Jin, Y. , Tian, Q. , Liu, Q. , Liu, W. , Zhang, C. , Lin, L. , Hu, Y. , Zhang, D. , Thu, M. , Zhang, Z. , Liu, S. , Zhong, C. , … Lai, Z. (2017). Genome‐wide sequencing of longan (*Dimocarpus longan* Lour.) provides insights into molecular basis of its polyphenol‐rich characteristics. GigaScience, 6, 1–14. 10.1093/gigascience/gix023 PMC5467034 28368449

[tpg220104-bib-0036] Liu, J. , Li, Y. , Wang, W. , Gai, J. , & Li, Y. (2016a). Genome‐wide analysis of MATE transporters and expression patterns of a subgroup of MATE genes in response to aluminum toxicity in soybean. BMC Genomics, 17, Article 223. 10.1186/s12864-016-2559-8 26968518 PMC4788864

[tpg220104-bib-0037] Liu, X. , Luo, Y. , Wu, H. , Xi, W. , Yu, J. , Zhang, Q. , & Zhou, Z. (2016b). Systematic analysis of O‐methyltransferase gene family and identification of potential members involved in the formation of O‐methylated flavonoids in Citrus. Gene, 575, 458–472. 10.1016/j.gene.2015.09.048 26407870

[tpg220104-bib-0038] Meyer, R. S. , & Purugganan, M. D. (2013). Evolution of crop species: Genetics of domestication and diversification. Nature Reviews Genetics, 14, 840–852. 10.1038/nrg3605 24240513

[tpg220104-bib-0039] Nevado, B. , Wong, E. L. Y. , Osborne, O. G. , & Filatov, D. A. (2019). Adaptive evolution is common in rapid evolutionary radiations. Current Biology, 29, 3081–3086.e5. 10.1016/j.cub.2019.07.059 31495580

[tpg220104-bib-0040] Nielsen, R. , Bustamante, C. , Clark, A. G. , Glanowski, S. , Sackton, T. B. , Hubisz, M. J. , Fledel‐Alon, A. , Tanenbaum, D. M. , Civello, D. , White, T. J. , Sninsky, J. , Adams, M. D. , & Cargill, M. (2005). A scan for positively selected genes in the genomes of humans and chimpanzees. PLOS Biology, 3. 10.1371/journal.pbio.0030170 PMC1088278 15869325

[tpg220104-bib-0041] Panchy, N. , Lehti‐Shiu, M. , & Shiu, S. ‐. H. (2016). Evolution of gene duplication in plants. Plant Physiology, 171, 2294–2316. 10.1104/pp.16.00523 27288366 PMC4972278

[tpg220104-bib-0042] Peaucelle, A. , Louvet, R. , Johansen, J. N. , Höfte, H. , Laufs, P. , Pelloux, J. , & Mouille, G. (2008). Arabidopsis phyllotaxis is controlled by the methyl‐esterification status of cell‐wall pectins. Current Biology, 18, 1943–1948. 10.1016/j.cub.2008.10.065 19097903

[tpg220104-bib-0043] Pelloux, J. , Rusterucci, C. , & Mellerowicz, E. (2007). New insights into pectin methylesterase structure and function. Trends in Plant Science, 12, 267–277. 10.1016/j.tplants.2007.04.001 17499007

[tpg220104-bib-0044] Pritchard, L. , White, J. A. , Birch, P. R. J. , & Toth, I. K. (2006). GenomeDiagram: A python package for the visualization of large‐scale genomic data. Bioinformatics, 22, 616–617. 10.1093/bioinformatics/btk021 16377612

[tpg220104-bib-0045] Qiao, X. , Li, Q. , Yin, H. , Qi, K. , Li, L. , Wang, R. , Zhang, S. , & Paterson, A. H. (2019). Gene duplication and evolution in recurring polyploidization–diploidization cycles in plants. Genome Biology, 20, 38. 10.1186/s13059-019-1650-2 30791939 PMC6383267

[tpg220104-bib-0046] Ren, R. , Wang, H. , Guo, C. , Zhang, N. , Zeng, L. , Chen, Y. , Ma, H. , & Qi, J.i (2018). Widespread whole genome duplications contribute to genome complexity and species diversity in angiosperms. Molecular Plant, 11, 414–428. 10.1016/j.molp.2018.01.002 29317285

[tpg220104-bib-0047] Santos, A. L. D. , Chaves‐Silva, S. , Yang, L. , Maia, L. G. S. , Chalfun‐Júnior, A. , Sinharoy, S. , Zhao, J. , & Benedito, V. A. (2017). Global analysis of the MATE gene family of metabolite transporters in tomato. BMC Plant Biology, 17. 10.1186/s12870-017-1115-2 PMC5663081 29084510

[tpg220104-bib-0048] Sawyer, S. L. , Wu, L. I. , Emerman, M. , & Malik, H. S. (2005). Positive selection of primate TRIM5α identifies a critical species‐specific retroviral restriction domain. Proceedings of the National Academy of Sciences of the United States of America, 102, 2832–2837. 10.1073/pnas.0409853102 15689398 PMC549489

[tpg220104-bib-0049] Springer, N. M. , Ying, K. , Fu, Y. , Ji, T. , Yeh, C. T. , Jia, Y.i , Wu, W. , Richmond, T. , Kitzman, J. , Rosenbaum, H. , Iniguez, A. L. , Barbazuk, W. B. , Jeddeloh, J. A. , Nettleton, D. , & Schnable, P. S. (2009). Maize inbreds exhibit high levels of copy number variation (CNV) and presence/absence variation (PAV) in genome content. PLOS Genetics, 5, 1–17. 10.1371/journal.pgen.1000734 PMC2780416 19956538

[tpg220104-bib-0050] Stamatakis, A. (2014). RAxML version 8: A tool for phylogenetic analysis and post‐analysis of large phylogenies. Bioinformatics, 30, 1312–1313. 10.1093/bioinformatics/btu033 24451623 PMC3998144

[tpg220104-bib-0051] Terol, J. , Ibañez, V. , Carbonell, J. , Alonso, R. , Estornell, L. H. , Licciardello, C. , Gut, I. G. , Dopazo, J. , & Talon, M. (2015). Involvement of a citrus meiotic recombination TTC‐repeat motif in the formation of gross deletions generated by ionizing radiation and MULE activation. BMC Genomics, 16, 69. 10.1186/s12864-015-1280-3 25758634 PMC4334395

[tpg220104-bib-0052] Terol, J. , Tadeo, F. , Ventimilla, D. , & Talon, M. (2016). An RNA‐Seq‐based reference transcriptome for Citrus. Plant Biotechnology Journal, 14, 938–950. 10.1111/pbi.12447 26261026 PMC11388863

[tpg220104-bib-0053] Van Bel, M. , Diels, T. , Vancaester, E. , Kreft, L. , Botzki, A. , Van De Peer, Y. , Coppens, F. , & Vandepoele, K. (2018). PLAZA 4.0: An integrative resource for functional, evolutionary and comparative plant genomics. Nucleic Acids Research, 46, D1190–D1196. 10.1093/nar/gkx1002 29069403 PMC5753339

[tpg220104-bib-0054] Auwera, G. A. , Carneiro, M. O. , Hartl, C. , Poplin, R. , Del Angel, G. , Levy‐Moonshine, A. , Jordan, T. , Shakir, K. , Roazen, D. , Thibault, J. , Banks, E. , Garimella, K. V. , Altshuler, D. , Gabriel, S. , & Depristo, M. A. (2013). From FastQ data to high confidence variant calls: The Genome Analysis Toolkit best practices pipeline. Current Protocols in Bioinformatics, 43, 11.10.1–11.10.33. 10.1002/0471250953.bi1110s43 PMC4243306 25431634

[tpg220104-bib-0055] Wang, L. , Lu, S. , Zhang, Y.e , Li, Z. , Du, X. , & Liu, D. (2014). Comparative genetic analysis of Arabidopsis purple acid phosphatases AtPAP10, AtPAP12, and AtPAP26 provides new insights into their roles in plant adaptation to phosphate deprivation. Journal of Integrative Plant Biology, 56, 299–314. 10.1111/jipb.12184 24528675

[tpg220104-bib-0056] Wang, S. , Yang, C. , Tu, H. , Zhou, J. , Liu, X. , Cheng, Y. , Luo, J. , Deng, X. , Zhang, H. , & Xu, J. (2017a). Characterization and metabolic diversity of flavonoids in citrus species. Scientific Reports, 7, 1–10. 10.1038/s41598-017-10970-2 28874745 PMC5585201

[tpg220104-bib-0057] Wang, X. , Xu, Y. , Zhang, S. , Cao, L.i , Huang, Y. , Cheng, J. , Wu, G. , Tian, S. , Chen, C. , Liu, Y. , Yu, H. , Yang, X. , Lan, H. , Wang, N. , Wang, L. , Xu, J. , Jiang, X. , Xie, Z. , Tan, M. , … Xu, Q. (2017b). Genomic analyses of primitive, wild and cultivated citrus provide insights into asexual reproduction. Nature Genetics, 49, 765–772. 10.1038/ng.3839 28394353

[tpg220104-bib-0058] Wu, G. A. , Prochnik, S. , Jenkins, J. , Salse, J. , Hellsten, U. , Murat, F. , Perrier, X. , Ruiz, M. , Scalabrin, S. , Terol, J. , Takita, M. A. , Labadie, K. , Poulain, J. , Couloux, A. , Jabbari, K. , Cattonaro, F. , Del Fabbro, C. , Pinosio, S. , Zuccolo, A. , … Rokhsar, D. (2014). Sequencing of diverse mandarin, pummelo and orange genomes reveals complex history of admixture during citrus domestication. Nature Biotechnology, 32, 656–662. 10.1038/nbt.2906 PMC4113729 24908277

[tpg220104-bib-0059] Wu, G. A. , Terol, J. , Ibanez, V. , López‐García, A. , Pérez‐Román, E. , Borredá, C. , Domingo, C. , Tadeo, F. R. , Carbonell‐Caballero, J. , Alonso, R. , Curk, F. , Du, D. , Ollitrault, P. , Roose, M. L. , Dopazo, J. , Gmitter, F. G. , Rokhsar, D. S. , & Talon, M. (2018). Genomics of the origin and evolution of *Citrus* . Nature, 554, 311–316. 10.1038/nature25447 29414943

[tpg220104-bib-0060] Wu, S. , Watanabe, N. , Mita, S. , Ueda, Y. , Shibuya, M. , & Ebizuka, Y. (2003). Two O‐methyltransferases isolated from flower petals of *Rosa chinensis* var. *spontanea* involved in scent biosynthesis. Journal of Bioscience and Bioengineering, 96, 119–128 10.1016/S1389-1723(03)90113-7 16233496

[tpg220104-bib-0061] Yang, Z. (2007). PAML 4: Phylogenetic analysis by maximum likelihood. Molecular Biology and Evolution, 24, 1586–1591. 10.1093/molbev/msm088 17483113

[tpg220104-bib-0062] Yuan, J. , Wang, J. , Yu, J. , Meng, F. , Zhao, Y. , Li, J. , Sun, P. , Sun, S. , Zhang, Z. , Liu, C. , Wei, C. , Guo, H.e , Li, X. , Duan, X. , Shen, S. , Xie, Y. , Hou, Y. , Zhang, J. , Shehzad, T. , & Wang, X. (2019). Alignment of Rutaceae genomes reveals lower genome fractionation level than Eudicot genomes affected by extra polyploidization. Frontiers in Plant Science, 10, 986. 10.3389/fpls.2019.00986 31447866 PMC6691040

[tpg220104-bib-0063] Zhang, J. (2003). Evolution of the human ASPM gene, a major determinant of brain size. Genetics, 165, 2063–2070 10.1093/genetics/165.4.2063 14704186 PMC1462882

[tpg220104-bib-0064] Zhang, R. , Zheng, F. , Wei, S. , Zhang, S. , Li, G. , Cao, P. , & Zhao, S. (2019). Evolution of disease defense genes and their regulators in plants. International Journal of Molecular Sciences, 20, 335. 10.3390/ijms20020335 30650550 PMC6358896

