# Supplementary material for: Shaping the biology of citrus: I. Genomic determinants of evolution
Source: Plant Genome. 2021 Jul 18;14(3):e20104. doi: 10.1002/tpg2.20104 (PMC12806996; doi:10.1002/tpg2.20104)
Supplement: Supplementary file 1 [file TPG2-14-e20104-s004.pdf]

Running title: Genomics of citrus evolution

Shaping the Biology of Citrus I: Genomic Determinants of Evolution

Daniel Gonzalez-Ibeas, Victoria Ibanez, Estela Perez-Roman, Carles Borredá, Javier Terol, Manuel Talon*

Affiliations: Valencian Institute of Agricultural Research (IVIA). Carretera Moncada CV-315, Km 10, 46113, Valencia, Spain.

*corresponding author: talon_man@gva.es

Supplementary figures.

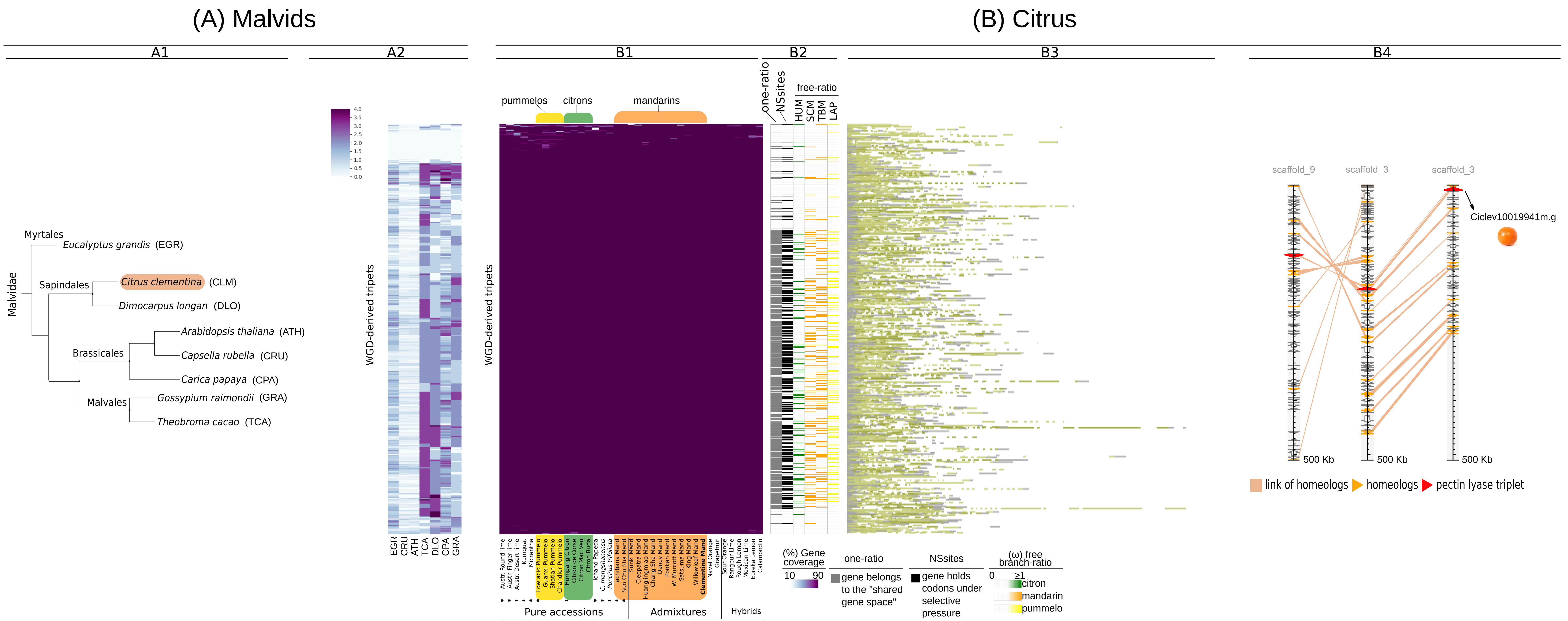

Figure S1. (A1) Phylogenetic tree of the Malvid clade represented by Citrus ssp. (Citrus clementine) and 7 additional species which were used for WGD comparison analysis. (A2) Presence/absence polymorphism of 612 citrus genes identified as WGD-derived triplets from the paleohexaploidy event in the 7 species of the Malvid clade. Gene count was normalized based on the number of recent lineage-specific WGDs experienced by each species: EGR 1 WGD, CRU and ATH 2 WGDs, GRA 1 WGD. (B1) Presence/absence polymorphism of the WGD-derived gene triplets (Y-axis, one gene per row) in different citrus accessions (X-axis), represented by a color scale of DNA read mapping coverage (color = gene present; white = gene absent). Genomes of pure citrus species used in the phylogenetic tree presented in Figure 1 are highlighted with an asterisk while the clementine reference is highlighted in bold. (B2) Codon selective pressure summary: gene belongs to the shared gene space (grey, 1st column); gene with codons identified under selective pressure by the NSsites codeml analysis (black, 2nd column); branch-omega value calculated by the free-ratio codeml analysis (columns 3-6) in citron (HUM, green), Tachibana (TBM, orange), Sun Chu Sha (SCM, orange) and pummelo (LAP, yellow). (B3) Representation of the exon/intron structure: green=exon, grey=UTR, empty=intron. Gene length is scaled to the longest one. (B4) Example of 1 triplet of 3 pectin lyases (red) within mandarin syntelogs from the paleohexaploidy event. Gene Ciclev10019941m.g was identified as potentially involved in citrus fruit size determination based on SNP data (see companion publication Gonzalez-Ibeas et al., 2021).

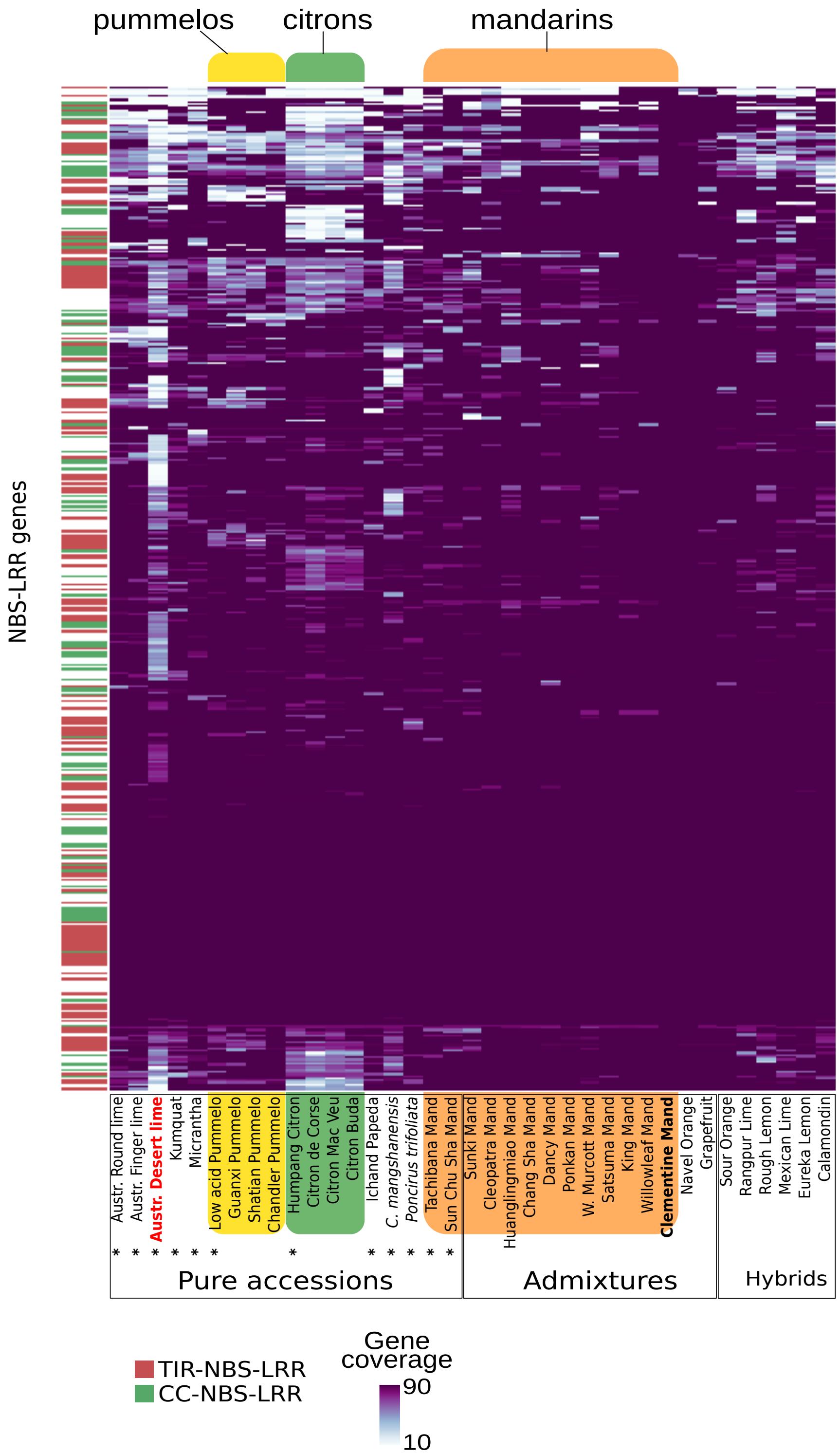

Figure S2. (A) Presence/absence polymorphism of pathogen-defense genes (Y-axis, one gene per row) in different citrus accessions (X-axis), represented by a color scale of DNA read mapping coverage (color = gene present; white = gene absent). Genomes of pure citrus species used as genomes of reference in the phylogenetic tree presented in Figure 1 are highlighted with an asterisk while the clementine reference is highlighted in bold. The Australian desert lime (*Eremocirus glauca*), that shows the highest number of polymorphisms in comparison with the genome of reference is highlighted in red. NBS-LRR pathogen defense genes where a TIR or CC protein domains can be identified are flagged in red and green, respectively.

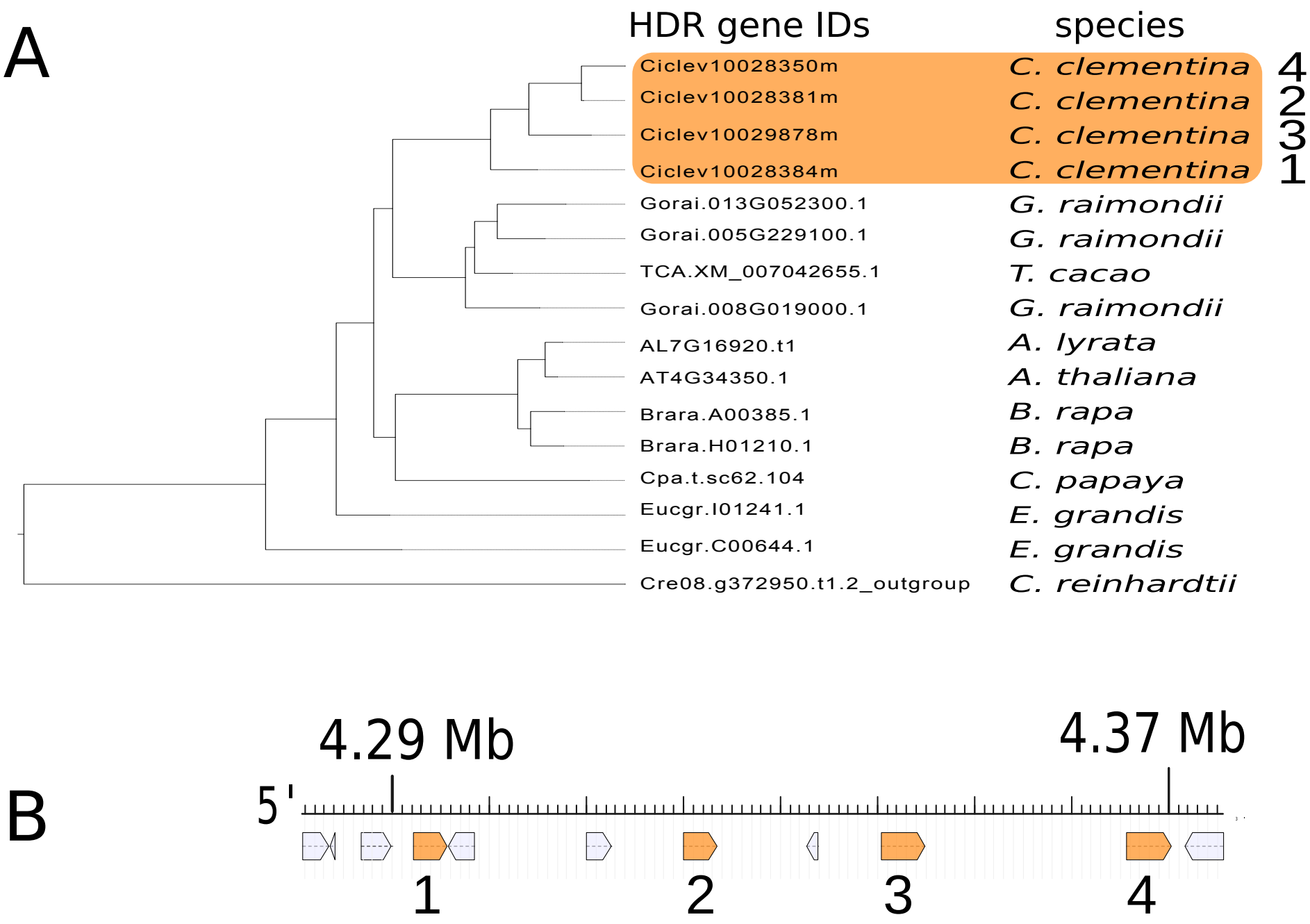

Figure S3. (A) Phylogenetic analysis of protein sequences of 1-hydroxy-2-methyl-2-(E)-butenyl-4-diphosphate reductase (*HDR*) genes from citrus and several species of the Malvidae clade. (B) Graphical representation of the four citrus *HDR* genes forming a cluster on chromosome 2.

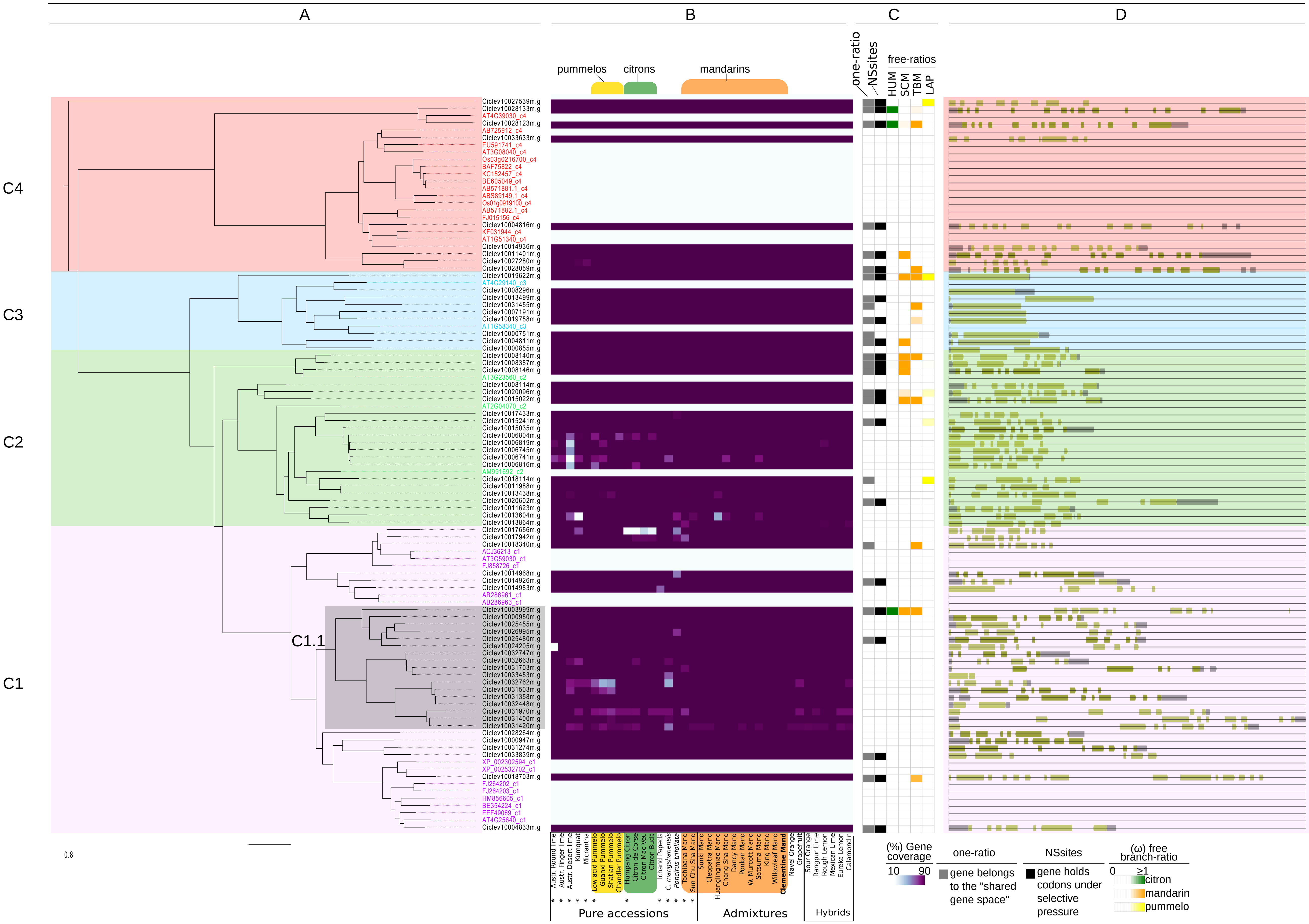

Figure S4. Analysis of *MATE-efflux* coding genes. (A) Phylogenetic analysis of 68 citrus MATE protein sequences (containing PF01554 domain) with 35 experimentally characterized MATE sequences from literature (J. Liu et al. 2016), classified into 4 families: C1=anthocyanin and flavonoid transport; C2=detoxification, C3=cell elongation; C4=detoxification. Each family cluster and the corresponding characterized gene accessions from literature are highlighted in 4 different colors. Citrus genes are written in black and the grey box highlights a subcluster of C1 family (C1.1) potentially specific of citrus. (B) Presence/absence polymorphism of citrus *MATE* genes (Y-axis, one gene per row) in different citrus accessions (X-axis), represented by a color scale of DNA read mapping coverage (color = gene present; white = gene absent). Genomes of pure citrus species used as genomes of reference in the phylogenetic tree presented in Figure 1 are highlighted with an asterisk while the clementine reference is highlighted in bold. (C) Codon selective pressure summary: gene belongs to the shared gene space (grey, 1st column); gene with codons identified under selective pressure by the NSsites codeml analysis (black, 2nd column); branch-omega value calculated by the free-ratio codeml analysis (columns 3-6) in citron (HUM, green), Tachibana (TBM, orange), Sun Chu Sha (SCM, orange) and pummelo (LAP, yellow). (D) Representation of the exon/intron structure: green=exon, grey=UTR, empty=intron. Gene length is scaled to the longest one. Intron/exon topology showed consistency among *MATE* families (C1-C4), being C2 mostly mono-exonic, C2 short intron-containing multiexonic, and C1 and C4 long intron-containing multiexonic.

Supplementary Tables.

Table S2.1 Gene Ontology (GO) categories overrepresented in genes with codons under selective pressure identified by the NSsites analysis (Adj. P-value < 0.05) Genes were ranked according to their number of codons identified under selective pressure and used as input for the logistic model of the Babelomics suite.

GO Category	adj P-value
metabolic process(GO:0008152)	1.39E-03
serine family amino acid metabolic process(GO:0009069)	9.77E-10
protein phosphorylation(GO:0006468)	9.65E-11
carbohydrate metabolic process(GO:0005975)	8.02E-03
signal transduction(GO:0007165)	4.74E-02
defense response(GO:0006952)	4.22E-14
peptidyl-tyrosine phosphorylation(GO:0018108)	5.17E-03
intracellular signal transduction(GO:0035556)	8.21E-04
protein targeting to membrane(GO:0006612)	2.47E-02
regulation of plant-type hypersensitive response(GO:0010363)	2.47E-02
transmembrane receptor protein tyrosine kinase signaling pathway(GO:0007169)	6.62E-04
drug transmembrane transport(GO:0006855)	2.89E-02
regulation of cellular process(GO:0050794)	1.18E-03
recognition of pollen(GO:0048544)	1.77E-04
cellular macromolecule metabolic process(GO:0044260)	6.28E-03
lipid catabolic process(GO:0016042)	1.11E-02
activation of MAPKK activity(GO:0000186)	7.53E-03
response to bacterium(GO:0009617)	1.92E-02
post-embryonic development(GO:0009791)	3.73E-04
organ development(GO:0048513)	2.38E-03
hexachlorocyclohexane metabolic process(GO:0019497)	4.69E-02
meiotic DNA double-strand break formation(GO:0042138)	1.85E-02
organic substance transport(GO:0071702)	6.43E-03
double-strand break repair via homologous recombination(GO:0000724)	1.26E-02
ion transmembrane transport(GO:0034220)	3.69E-02
regulation of cell proliferation(GO:0042127)	3.72E-02
mRNA export from nucleus(GO:0006406)	1.02E-02
protein desumoylation(GO:0016926)	1.14E-02
response to insect(GO:0009625)	2.16E-02
cellular protein metabolic process(GO:0044267)	4.38E-02
detection of biotic stimulus(GO:0009595)	4.71E-02
lignin catabolic process(GO:0046274)	9.60E-04
regulation of macromolecule metabolic process(GO:0060255)	1.18E-03
innate immune response(GO:0045087)	2.41E-03
oxylipin biosynthetic process(GO:0031408)	5.34E-03
cellular protein modification process(GO:0006464)	4.17E-02
reproductive process(GO:0022414)	6.55E-03
pectin catabolic process(GO:0045490)	2.22E-02
meiosis I(GO:0007127)	2.29E-03
taurine metabolic process(GO:0019530)	4.44E-02
defense response by callose deposition(GO:0052542)	1.72E-04
reproductive structure development(GO:0048608)	2.45E-04
regionalization(GO:0003002)	1.96E-02
regulation of stomatal closure(GO:0090333)	1.96E-02
floral organ development(GO:0048437)	2.61E-02
lipid oxidation(GO:0034440)	1.28E-04
defense response by callose deposition in cell wall(GO:0052544)	7.82E-04
shade avoidance(GO:0009641)	1.21E-03
xylan catabolic process(GO:0045493)	3.26E-02

glutathione catabolic process(GO:0006751)	4.24E-03
response to herbivore(GO:0080027)	6.14E-03
RNA-directed DNA methylation(GO:0080188)	3.47E-03
stomatal closure(GO:0090332)	1.57E-02
leukotriene metabolic process(GO:0006691)	2.47E-02
prostaglandin metabolic process(GO:0006693)	2.47E-02
mismatch repair(GO:0006298)	2.61E-02
ion transport(GO:0006811)	3.26E-02
phenylpropanoid biosynthetic process(GO:0009699)	3.64E-02
macromolecule modification(GO:0043412)	2.52E-05
regulation of DNA methylation(GO:0044030)	3.19E-05
induced systemic resistance(GO:0009682)	8.87E-04
alanyl-tRNA aminoacylation(GO:0006419)	5.96E-03
oogenesis(GO:0048477)	7.13E-03
L-arabinose metabolic process(GO:0046373)	1.02E-02
manganese ion transport(GO:0006828)	2.11E-02
cell plate formation involved in plant-type cell wall biogenesis(GO:0009920)	4.35E-02
cGMP biosynthetic process(GO:0006182)	2.88E-04
GTP metabolic process(GO:0046039)	2.88E-04
regulation of ethylene biosynthetic process(GO:0010364)	1.54E-03
detection of external stimulus(GO:0009581)	1.54E-03
cellular response to indolebutyric acid stimulus(GO:0071366)	6.87E-03
phosphatidylcholine metabolic process(GO:0046470)	1.01E-02
negative regulation of defense response to bacterium(GO:1900425)	1.50E-02
xenobiotic metabolic process(GO:0006805)	1.50E-02
detection of chemical stimulus(GO:0009593)	1.50E-02
detection of external biotic stimulus(GO:0098581)	1.50E-02
Golgi to vacuole transport(GO:0006896)	1.50E-02
glucosinolate catabolic process(GO:0019762)	2.24E-02
regulation of synergid differentiation(GO:0045697)	3.33E-02
clathrin-mediated endocytosis(GO:0072583)	3.33E-02
protein localization to plasma membrane(GO:0072659)	4.97E-02
response to manganese ion(GO:0010042)	4.97E-02
cellular manganese ion homeostasis(GO:0030026)	4.97E-02
indole glucosinolate biosynthetic process(GO:0009759)	1.83E-04
histone H3-K18 acetylation(GO:0043971)	4.22E-04
histone H3-K23 acetylation(GO:0043972)	4.22E-04
histone H3-K14 acetylation(GO:0044154)	4.22E-04
negative regulation of secondary growth(GO:2000604)	5.48E-04
stem cell division(GO:0017145)	9.80E-04
regulation of post-embryonic root development(GO:2000069)	9.80E-04
translesion synthesis(GO:0019985)	1.89E-03
trehalose catabolic process(GO:0005993)	4.75E-03
regulation of DNA demethylation(GO:1901535)	4.75E-03
positive regulation of sister chromatid cohesion(GO:0045876)	5.79E-03
meiotic chromosome separation(GO:0051307)	5.79E-03
superoxide anion generation(GO:0042554)	7.08E-03
xanthine metabolic process(GO:0046110)	7.08E-03
histone H3-K9 demethylation(GO:0033169)	1.32E-02
negative regulation of short-day photoperiodism, flowering(GO:0048577)	1.32E-02
negative regulation of long-day photoperiodism, flowering(GO:0048579)	1.32E-02
negative regulation of defense response to insect(GO:1900366)	2.04E-02
putrescine biosynthetic process from ornithine(GO:0033387)	3.18E-02
response to photooxidative stress(GO:0080183)	3.18E-02
positive regulation of cell fate specification(GO:0042660)	3.18E-02
regulation of histone H3-K9 dimethylation(GO:1900109)	4.58E-02

Table S2.2 Gene Ontology (GO) categories overrepresented in genes with codons under selective pressure identified by the NSsites analysis (Adj. P-value < 0.05) Genes were ranked according to their adjusted P-values derived from the NSsites test and used as input for the logistic model of the Babelomics suite

GO Category	adj P-value
oxidation-reduction process(GO:0055114)	2.11E-02
obsolete ATP catabolic process(GO:0006200)	4.24E-02
protein phosphorylation(GO:0006468)	4.30E-04
carbohydrate metabolic process(GO:0005975)	2.80E-02
transmembrane transport(GO:0055085)	1.43E-02
serine family amino acid metabolic process(GO:0009069)	1.99E-05
vegetative to reproductive phase transition of meristem(GO:0010228)	4.44E-02
starch metabolic process(GO:0005982)	8.80E-04
sucrose metabolic process(GO:0005985)	8.79E-04
glycerolipid metabolic process(GO:0046486)	1.12E-02
cellulose biosynthetic process(GO:0030244)	2.05E-02
peptidyl-tyrosine phosphorylation(GO:0018108)	1.18E-02
chloroplast relocation(GO:0009902)	4.67E-02
transmembrane receptor protein tyrosine kinase signaling pathway(GO:0007169)	1.72E-02
microtubule-based movement(GO:0007018)	4.49E-02

Table S3. List of genes identified as WGD-derived triplets from the paleohexaploidy shared by all eudicots.

Gene ID 1	Gene ID 2	Gene ID 3	Functional annotation
Ciclev10009889m.v1.0	Ciclev10016599m.v1.0	Ciclev10016648m.v1.0	B-cell receptor-associated 31-like
Ciclev10012000m.v1.0	Ciclev10028738m.v1.0	Ciclev10031679m.v1.0	zinc finger (C3HC4-type RING finger) family protein
Ciclev10011501m.v1.0	Ciclev10019383m.v1.0	Ciclev10031162m.v1.0	WRKY DNA-binding protein 72
Ciclev10005899m.v1.0	Ciclev10022286m.v1.0	Ciclev10023664m.v1.0	Remorin family protein
Ciclev10006217m.v1.0	Ciclev10022145m.v1.0	Ciclev10022936m.v1.0	AGAMOUS-like 42
Ciclev10001015m.v1.0	Ciclev10025331m.v1.0	Ciclev10028350m.v1.0	DA1
Ciclev10008349m.v1.0	Ciclev10015634m.v1.0	Ciclev10015687m.v1.0	Plant protein of unknown function (DUF828)
Ciclev10009447m.v1.0	Ciclev10009526m.v1.0	Ciclev10027305m.v1.0	Integrase-type DNA-binding superfamily protein
Ciclev10014804m.v1.0	Ciclev10019547m.v1.0	Ciclev10031145m.v1.0	AMP-dependent synthetase and ligase family protein
Ciclev10003620m.v1.0	Ciclev10026862m.v1.0	Ciclev10029669m.v1.0	RAD-like 6
Ciclev10012970m.v1.0	Ciclev10029407m.v1.0	Ciclev10033996m.v1.0	no_ann
Ciclev10011622m.v1.0	Ciclev10019935m.v1.0	Ciclev10031390m.v1.0	UDP-glucose 6-dehydrogenase family protein
Ciclev10012518m.v1.0	Ciclev10029094m.v1.0	Ciclev10032484m.v1.0	expansin A4
Ciclev10006487m.v1.0	Ciclev10022942m.v1.0	Ciclev10023137m.v1.0	no_ann
Ciclev10003698m.v1.0	Ciclev10006161m.v1.0	Ciclev10022856m.v1.0	GATA transcription factor 15
Ciclev10000364m.v1.0	Ciclev10024929m.v1.0	Ciclev10027850m.v1.0	Subtilisin-like serine endopeptidase family protein
Ciclev10017502m.v1.0	Ciclev10022482m.v1.0	Ciclev10032807m.v1.0	ADP-ribosylation factor A1B
Ciclev10020725m.v1.0	Ciclev10021521m.v1.0	Ciclev10021862m.v1.0	Dof-type zinc finger DNA-binding family protein
Ciclev10011783m.v1.0	Ciclev10019832m.v1.0	Ciclev10031172m.v1.0	no_ann
Ciclev10009138m.v1.0	Ciclev10009370m.v1.0	Ciclev10027587m.v1.0	Integrase-type DNA-binding superfamily protein
Ciclev10007908m.v1.0	Ciclev10008610m.v1.0	Ciclev10025314m.v1.0	homology to ABI1
Ciclev10005220m.v1.0	Ciclev10021073m.v1.0	Ciclev10021422m.v1.0	cytokinin response factor 4
Ciclev10011556m.v1.0	Ciclev10019949m.v1.0	Ciclev10031433m.v1.0	cycling DOF factor 2
Ciclev10011179m.v1.0	Ciclev10014231m.v1.0	Ciclev10024220m.v1.0	Protein kinase superfamily protein
Ciclev10001979m.v1.0	Ciclev10026107m.v1.0	Ciclev10029124m.v1.0	myb domain protein r1
Ciclev10008439m.v1.0	Ciclev10015441m.v1.0	Ciclev10015443m.v1.0	Galactosyltransferase family protein
Ciclev10009828m.v1.0	Ciclev10017087m.v1.0	Ciclev10017871m.v1.0	no_ann
Ciclev10012295m.v1.0	Ciclev10030209m.v1.0	Ciclev10033532m.v1.0	zinc-finger protein 10
Ciclev10006549m.v1.0	Ciclev10019647m.v1.0	Ciclev10023948m.v1.0	Arabidopsis thaliana protein of unknown function (DUF821)
Ciclev10006155m.v1.0	Ciclev10022708m.v1.0	Ciclev10022714m.v1.0	ubiquitin-conjugating enzyme 10
Ciclev10010985m.v1.0	Ciclev10028453m.v1.0	Ciclev10033801m.v1.0	KCBP-interacting protein kinase
Ciclev10016434m.v1.0	Ciclev10032547m.v1.0	Ciclev10033415m.v1.0	NAC domain containing protein 84
Ciclev10000827m.v1.0	Ciclev10025176m.v1.0	Ciclev10028023m.v1.0	Sec14p-like phosphatidylinositol transfer family protein
Ciclev10011194m.v1.0	Ciclev10027901m.v1.0	Ciclev10030860m.v1.0	auxin response factor 16
Ciclev10013311m.v1.0	Ciclev10029464m.v1.0	Ciclev10032889m.v1.0	C2H2 and C2HC zinc fingers superfamily protein
Ciclev10013147m.v1.0	Ciclev10017988m.v1.0	Ciclev10029587m.v1.0	no_ann
Ciclev10011996m.v1.0	Ciclev10020723m.v1.0	Ciclev10031914m.v1.0	reversibly glycosylated polypeptide 1
Ciclev10011270m.v1.0	Ciclev10019356m.v1.0	Ciclev10030931m.v1.0	phosphoinositide 4-kinase gamma 7
Ciclev10005557m.v1.0	Ciclev10021409m.v1.0	Ciclev10021489m.v1.0	phloem protein 2-A13
Ciclev10009918m.v1.0	Ciclev10009919m.v1.0	Ciclev10026972m.v1.0	Mitochondrial ATP synthase subunit G protein
Ciclev10015213m.v1.0	Ciclev10024278m.v1.0	Ciclev10033376m.v1.0	lysine histidine transporter 1
Ciclev10009879m.v1.0	Ciclev10016841m.v1.0	Ciclev10017104m.v1.0	squamosa promoter binding protein-like 5
Ciclev10011218m.v1.0	Ciclev10030201m.v1.0	Ciclev10030854m.v1.0	receptor lectin kinase
Ciclev10012375m.v1.0	Ciclev10028975m.v1.0	Ciclev10032302m.v1.0	plasma membrane intrinsic protein 2
Ciclev10000155m.v1.0	Ciclev10026946m.v1.0	Ciclev10027748m.v1.0	Leucine-rich receptor-like protein kinase family protein
Ciclev10001824m.v1.0	Ciclev10025993m.v1.0	Ciclev10029805m.v1.0	SHI-related sequence 5
Ciclev10017392m.v1.0	Ciclev10033156m.v1.0	Ciclev10033268m.v1.0	Protein of unknown function (DUF 3339)
Ciclev10007831m.v1.0	Ciclev10010641m.v1.0	Ciclev10025189m.v1.0	Major facilitator superfamily protein
Ciclev10003991m.v1.0	Ciclev10004297m.v1.0	Ciclev10024513m.v1.0	no_ann
Ciclev10002290m.v1.0	Ciclev10005789m.v1.0	Ciclev10022032m.v1.0	indoleacetic acid-induced protein 16
Ciclev10001429m.v1.0	Ciclev10025674m.v1.0	Ciclev10030135m.v1.0	branched-chain amino acid transaminase 2
Ciclev10017762m.v1.0	Ciclev10024093m.v1.0	Ciclev10031917m.v1.0	Leucine-rich repeat protein kinase family protein

Ciclev10015945m.v1.0	Ciclev10021025m.v1.0	Ciclev10032350m.v1.0	Protein of unknown function (DUF1645)
Ciclev10009922m.v1.0	Ciclev10017018m.v1.0	Ciclev10017200m.v1.0	Ubiquitin-like superfamily protein
Ciclev10005820m.v1.0	Ciclev10023546m.v1.0	Ciclev10024142m.v1.0	ethylene response factor 1
Ciclev10011108m.v1.0	Ciclev10027794m.v1.0	Ciclev10030911m.v1.0	pseudo-response regulator 7
Ciclev10012024m.v1.0	Ciclev10028829m.v1.0	Ciclev10032039m.v1.0	DNA binding with one finger 2.4
Ciclev10011676m.v1.0	Ciclev10020038m.v1.0	Ciclev10031630m.v1.0	Nuclear transport factor 2 (NTF2) family protein with RNA binding (RRM-RBD-RNP motifs) domain
Ciclev10011882m.v1.0	Ciclev10021061m.v1.0	Ciclev10031813m.v1.0	ABI five binding protein 3
Ciclev10013705m.v1.0	Ciclev10016422m.v1.0	Ciclev10022068m.v1.0	LOB domain-containing protein 41
Ciclev10011539m.v1.0	Ciclev10028249m.v1.0	Ciclev10031288m.v1.0	HSP20-like chaperones superfamily protein
Ciclev10012390m.v1.0	Ciclev10021514m.v1.0	Ciclev10032213m.v1.0	SPFH/Band 7/PHB domain-containing membrane-associated protein family
Ciclev10017477m.v1.0	Ciclev10021203m.v1.0	Ciclev10023449m.v1.0	no_ann
Ciclev10009041m.v1.0	Ciclev10009083m.v1.0	Ciclev10027065m.v1.0	SPX domain gene 2
Ciclev10013809m.v1.0	Ciclev10028161m.v1.0	Ciclev10031031m.v1.0	Ankyrin repeat family protein
Ciclev10012135m.v1.0	Ciclev10020248m.v1.0	Ciclev10032020m.v1.0	BES1-interacting Myc-like protein 3
Ciclev10009080m.v1.0	Ciclev10009255m.v1.0	Ciclev10026074m.v1.0	alpha/beta-Hydrolases superfamily protein
Ciclev10011890m.v1.0	Ciclev10028876m.v1.0	Ciclev10031854m.v1.0	Protein kinase superfamily protein
Ciclev10007178m.v1.0	Ciclev10022285m.v1.0	Ciclev10024438m.v1.0	Plant invertase/pectin methylesterase inhibitor superfamily protein
Ciclev10005144m.v1.0	Ciclev10015805m.v1.0	Ciclev10032206m.v1.0	nuclear factor Y, subunit A1
Ciclev10000260m.v1.0	Ciclev10024881m.v1.0	Ciclev10030257m.v1.0	beta-galactosidase 3
Ciclev10004423m.v1.0	Ciclev10014436m.v1.0	Ciclev10030816m.v1.0	CCCH-type zinc finger protein with ARM repeat domain
Ciclev10011134m.v1.0	Ciclev10027912m.v1.0	Ciclev10030821m.v1.0	PHE ammonia lyase 1
Ciclev10011992m.v1.0	Ciclev10029989m.v1.0	Ciclev10032941m.v1.0	myb-like HTH transcriptional regulator family protein
Ciclev10009957m.v1.0	Ciclev10010422m.v1.0	Ciclev10017223m.v1.0	Calcium-binding EF-hand family protein
Ciclev10002562m.v1.0	Ciclev10026554m.v1.0	Ciclev10029323m.v1.0	endoplasmatic reticulum retrieval protein 1B
Ciclev10003746m.v1.0	Ciclev10026374m.v1.0	Ciclev10029274m.v1.0	LOB domain-containing protein 39
Ciclev10013407m.v1.0	Ciclev10028253m.v1.0	Ciclev10033768m.v1.0	cytochrome P450, family 94, subfamily C, polypeptide 1
Ciclev10017531m.v1.0	Ciclev10032283m.v1.0	Ciclev10032943m.v1.0	WRKY DNA-binding protein 75
Ciclev10012594m.v1.0	Ciclev10021822m.v1.0	Ciclev10032572m.v1.0	AGAMOUS-like 8
Ciclev10013181m.v1.0	Ciclev10017614m.v1.0	Ciclev10023053m.v1.0	Bifunctional inhibitor/lipid-transfer protein/seed storage 2S albumin superfamily protein
Ciclev10009189m.v1.0	Ciclev10009202m.v1.0	Ciclev10025712m.v1.0	Tetratricopeptide repeat (TPR)-like superfamily protein
Ciclev10015110m.v1.0	Ciclev10031521m.v1.0	Ciclev10031571m.v1.0	ACT domain repeat 6
Ciclev10012908m.v1.0	Ciclev10016881m.v1.0	Ciclev10022616m.v1.0	no_ann
Ciclev10003219m.v1.0	Ciclev10026305m.v1.0	Ciclev10029910m.v1.0	no_ann
Ciclev10001054m.v1.0	Ciclev10025887m.v1.0	Ciclev10028338m.v1.0	Granulin repeat cysteine protease family protein
Ciclev10010892m.v1.0	Ciclev10027676m.v1.0	Ciclev10030474m.v1.0	demeter-like 1
Ciclev10007886m.v1.0	Ciclev10008181m.v1.0	Ciclev10025255m.v1.0	phosphofructokinase 3
Ciclev10006544m.v1.0	Ciclev10020705m.v1.0	Ciclev10023950m.v1.0	RING/U-box superfamily protein
Ciclev10007087m.v1.0	Ciclev10023304m.v1.0	Ciclev10023747m.v1.0	actin depolymerizing factor 7
Ciclev10022978m.v1.0	Ciclev10022980m.v1.0	Ciclev10022987m.v1.0	Yippee family putative zinc-binding protein
Ciclev10012557m.v1.0	Ciclev10017573m.v1.0	Ciclev10023387m.v1.0	no_ann
Ciclev10001177m.v1.0	Ciclev10025699m.v1.0	Ciclev10028539m.v1.0	Protein kinase superfamily protein
Ciclev10017531m.v1.0	Ciclev10032816m.v1.0	Ciclev10032943m.v1.0	WRKY DNA-binding protein 75
Ciclev10007917m.v1.0	Ciclev10014642m.v1.0	Ciclev10014665m.v1.0	WRKY family transcription factor
Ciclev10005165m.v1.0	Ciclev10020744m.v1.0	Ciclev10021038m.v1.0	WRKY family transcription factor
Ciclev10011779m.v1.0	Ciclev10028623m.v1.0	Ciclev10031403m.v1.0	Plant protein of unknown function (DUF828)
Ciclev10001235m.v1.0	Ciclev10025564m.v1.0	Ciclev10028938m.v1.0	CYCLIN B2;4
Ciclev10007724m.v1.0	Ciclev10014193m.v1.0	Ciclev10014998m.v1.0	Ubiquitin-conjugating enzyme family protein
Ciclev10004864m.v1.0	Ciclev10019597m.v1.0	Ciclev10019941m.v1.0	Pectin lyase-like superfamily protein
Ciclev10002418m.v1.0	Ciclev10005825m.v1.0	Ciclev10022082m.v1.0	Drought-responsive family protein
Ciclev10012445m.v1.0	Ciclev10021785m.v1.0	Ciclev10032366m.v1.0	voltage dependent anion channel 1
Ciclev10011561m.v1.0	Ciclev10019977m.v1.0	Ciclev10031223m.v1.0	C2H2-like zinc finger protein
Ciclev10011588m.v1.0	Ciclev10028259m.v1.0	Ciclev10031306m.v1.0	Integrase-type DNA-binding superfamily protein
Ciclev10012928m.v1.0	Ciclev10029402m.v1.0	Ciclev10032972m.v1.0	Family of unknown function (DUF662)
Ciclev10002819m.v1.0	Ciclev10006193m.v1.0	Ciclev10022807m.v1.0	Ribosomal protein S24e family protein
Ciclev10008027m.v1.0	Ciclev10014941m.v1.0	Ciclev10017754m.v1.0	aspartic proteinase A1
Ciclev10005981m.v1.0	Ciclev10022206m.v1.0	Ciclev10022440m.v1.0	regulatory components of ABA receptor 3

Ciclev10016166m.v1.0	Ciclev10024575m.v1.0	Ciclev10032514m.v1.0	carbonic anhydrase 1
Ciclev10000581m.v1.0	Ciclev10006445m.v1.0	Ciclev10024255m.v1.0	cysteine-rich RLK (RECEPTOR-like protein kinase) 10
Ciclev10011948m.v1.0	Ciclev10023440m.v1.0	Ciclev10033327m.v1.0	myb domain protein 106
Ciclev10000839m.v1.0	Ciclev10025367m.v1.0	Ciclev10029886m.v1.0	FAD/NAD(P)-binding oxidoreductase family protein
Ciclev10011507m.v1.0	Ciclev10018999m.v1.0	Ciclev10031011m.v1.0	Pentatricopeptide repeat (PPR) superfamily protein
Ciclev10009203m.v1.0	Ciclev10016218m.v1.0	Ciclev10016298m.v1.0	membrane-associated mannitol-induced
Ciclev10011115m.v1.0	Ciclev10027998m.v1.0	Ciclev10030942m.v1.0	exocyst subunit exo70 family protein H4
Ciclev10005901m.v1.0	Ciclev10022288m.v1.0	Ciclev10022295m.v1.0	Protein of unknown function (DUF640)
Ciclev10012988m.v1.0	Ciclev10029428m.v1.0	Ciclev10033601m.v1.0	HSP20-like chaperones superfamily protein
Ciclev10001043m.v1.0	Ciclev10027226m.v1.0	Ciclev10028355m.v1.0	cytochrome P450, family 707, subfamily A, polypeptide 4
Ciclev10005787m.v1.0	Ciclev10018158m.v1.0	Ciclev10032575m.v1.0	related to AP2 6l
Ciclev10011968m.v1.0	Ciclev10023399m.v1.0	Ciclev10032160m.v1.0	homeobox protein 34
Ciclev10012221m.v1.0	Ciclev10029069m.v1.0	Ciclev10032389m.v1.0	NAD(P)-binding Rossmann-fold superfamily protein
Ciclev10002897m.v1.0	Ciclev10006201m.v1.0	Ciclev10023750m.v1.0	ralf-like 33
Ciclev10002314m.v1.0	Ciclev10025376m.v1.0	Ciclev10028399m.v1.0	AP2/B3-like transcriptional factor family protein
Ciclev10009673m.v1.0	Ciclev10009702m.v1.0	Ciclev10026612m.v1.0	no_ann
Ciclev10011463m.v1.0	Ciclev10028378m.v1.0	Ciclev10030855m.v1.0	no_ann
Ciclev10013811m.v1.0	Ciclev10015505m.v1.0	Ciclev10020320m.v1.0	Core-2/I-branching beta-1,6-N-acetylglucosaminyltransferase family protein
Ciclev10017767m.v1.0	Ciclev10021037m.v1.0	Ciclev10032014m.v1.0	glyceraldehyde-3-phosphate dehydrogenase C subunit 1
Ciclev10003170m.v1.0	Ciclev10026015m.v1.0	Ciclev10026166m.v1.0	cyclin d5;1
Ciclev10012453m.v1.0	Ciclev10016461m.v1.0	Ciclev10024155m.v1.0	BR enhanced expression 3
Ciclev10002761m.v1.0	Ciclev10006129m.v1.0	Ciclev10021954m.v1.0	histidine-containing phosphotransmitter 1
Ciclev10009760m.v1.0	Ciclev10009893m.v1.0	Ciclev10026703m.v1.0	Ran BP2/NZF zinc finger-like superfamily protein
Ciclev10012730m.v1.0	Ciclev10021492m.v1.0	Ciclev10032321m.v1.0	RING/FYVE/PHD zinc finger superfamily protein
Ciclev10011455m.v1.0	Ciclev10028164m.v1.0	Ciclev10031284m.v1.0	Nucleotide-diphospho-sugar transferases superfamily protein
Ciclev10013507m.v1.0	Ciclev10027927m.v1.0	Ciclev10030878m.v1.0	TRF-like 9
Ciclev10011140m.v1.0	Ciclev10027848m.v1.0	Ciclev10030787m.v1.0	GRAS family transcription factor
Ciclev10000934m.v1.0	Ciclev10000967m.v1.0	Ciclev10025420m.v1.0	cytochrome P450, family 81, subfamily D, polypeptide 8
Ciclev10010150m.v1.0	Ciclev10014400m.v1.0	Ciclev10017595m.v1.0	mitogen-activated protein kinase kinase kinase 15
Ciclev10012704m.v1.0	Ciclev10023416m.v1.0	Ciclev10033684m.v1.0	no_ann
Ciclev10009223m.v1.0	Ciclev10014447m.v1.0	Ciclev10017664m.v1.0	Concanavalin A-like lectin protein kinase family protein
Ciclev10018177m.v1.0	Ciclev10023699m.v1.0	Ciclev10030967m.v1.0	Protein kinase family protein with domain of unknown function (DUF26)
Ciclev10008000m.v1.0	Ciclev10010400m.v1.0	Ciclev10027246m.v1.0	calmodulin-binding family protein
Ciclev10011900m.v1.0	Ciclev10015256m.v1.0	Ciclev10031573m.v1.0	Emsy N Terminus (ENT)/ plant Tudor-like domains-containing protein
Ciclev10008157m.v1.0	Ciclev10015084m.v1.0	Ciclev10015098m.v1.0	RNA-binding (RRM/RBD/RNP motifs) family protein
Ciclev10011358m.v1.0	Ciclev10028036m.v1.0	Ciclev10031134m.v1.0	laccase 17
Ciclev10012285m.v1.0	Ciclev10028910m.v1.0	Ciclev10032309m.v1.0	Duplicated homeodomain-like superfamily protein
Ciclev10010462m.v1.0	Ciclev10010639m.v1.0	Ciclev10027032m.v1.0	allergen-related
Ciclev10009050m.v1.0	Ciclev10016303m.v1.0	Ciclev10018064m.v1.0	MYB-like 102
Ciclev10002566m.v1.0	Ciclev10005438m.v1.0	Ciclev10022379m.v1.0	secretion-associated RAS super family 2
Ciclev10012611m.v1.0	Ciclev10021897m.v1.0	Ciclev10032545m.v1.0	expansin A5
Ciclev10011211m.v1.0	Ciclev10020197m.v1.0	Ciclev10020517m.v1.0	cysteine-rich RLK (RECEPTOR-like protein kinase) 20
Ciclev10003614m.v1.0	Ciclev10025861m.v1.0	Ciclev10028762m.v1.0	P-loop containing nucleoside triphosphate hydrolases superfamily protein
Ciclev10009512m.v1.0	Ciclev10009884m.v1.0	Ciclev10026799m.v1.0	Protein of unknown function (DUF3511)
Ciclev10001416m.v1.0	Ciclev10025890m.v1.0	Ciclev10028675m.v1.0	CYCLIN D3;1
Ciclev10005629m.v1.0	Ciclev10021699m.v1.0	Ciclev10022057m.v1.0	myb domain protein 15
Ciclev10012125m.v1.0	Ciclev10028790m.v1.0	Ciclev10031933m.v1.0	Cysteine proteinases superfamily protein
Ciclev10012725m.v1.0	Ciclev10029257m.v1.0	Ciclev10032714m.v1.0	Ras-related small GTP-binding family protein
Ciclev10007668m.v1.0	Ciclev10007810m.v1.0	Ciclev10025125m.v1.0	NADP-malic enzyme 3
Ciclev10011980m.v1.0	Ciclev10015803m.v1.0	Ciclev10030362m.v1.0	alpha/beta-Hydrolases superfamily protein
Ciclev10013312m.v1.0	Ciclev10014485m.v1.0	Ciclev10033813m.v1.0	DNA-binding bromodomain-containing protein
Ciclev10013334m.v1.0	Ciclev10024105m.v1.0	Ciclev10032777m.v1.0	basic leucine-zipper 42
Ciclev10010488m.v1.0	Ciclev10010629m.v1.0	Ciclev10027153m.v1.0	gibberellin 3-oxidase 3
Ciclev10013131m.v1.0	Ciclev10022900m.v1.0	Ciclev10033093m.v1.0	Ribosomal protein L34e superfamily protein
Ciclev10003157m.v1.0	Ciclev10006769m.v1.0	Ciclev10024294m.v1.0	nuclear factor Y, subunit B4
Ciclev10011638m.v1.0	Ciclev10023704m.v1.0	Ciclev10031645m.v1.0	Protein kinase superfamily protein

Ciclev10012093m.v1.0	Ciclev10020480m.v1.0	Ciclev10032304m.v1.0	NAC domain containing protein 100
Ciclev10004478m.v1.0	Ciclev10019056m.v1.0	Ciclev10019076m.v1.0	Leucine-rich repeat protein kinase family protein
Ciclev10007188m.v1.0	Ciclev10019040m.v1.0	Ciclev10019694m.v1.0	Protein kinase superfamily protein
Ciclev10000537m.v1.0	Ciclev10025109m.v1.0	Ciclev10027999m.v1.0	poly(A) binding protein 8
Ciclev10001552m.v1.0	Ciclev10025588m.v1.0	Ciclev10028426m.v1.0	Tubulin/FtsZ family protein
Ciclev10012612m.v1.0	Ciclev10024246m.v1.0	Ciclev10033382m.v1.0	TCP family transcription factor
Ciclev10005095m.v1.0	Ciclev10020943m.v1.0	Ciclev10024061m.v1.0	WRKY DNA-binding protein 27
Ciclev10013568m.v1.0	Ciclev10028100m.v1.0	Ciclev10030988m.v1.0	Major facilitator superfamily protein
Ciclev10002551m.v1.0	Ciclev10026539m.v1.0	Ciclev10029311m.v1.0	RAC-like 3
Ciclev10003671m.v1.0	Ciclev10027113m.v1.0	Ciclev10028320m.v1.0	purple acid phosphatase 12
Ciclev10012786m.v1.0	Ciclev10022925m.v1.0	Ciclev10033115m.v1.0	Gibberellin-regulated family protein
Ciclev10007927m.v1.0	Ciclev10014811m.v1.0	Ciclev10014845m.v1.0	scarecrow-like 5
Ciclev10002567m.v1.0	Ciclev10025075m.v1.0	Ciclev10028128m.v1.0	no_ann
Ciclev10010577m.v1.0	Ciclev10017523m.v1.0	Ciclev10018204m.v1.0	glutamate receptor 2.1
Ciclev10012700m.v1.0	Ciclev10022140m.v1.0	Ciclev10032709m.v1.0	Vesicle transport v-SNARE family protein
Ciclev10006897m.v1.0	Ciclev10020967m.v1.0	Ciclev10021268m.v1.0	myb domain protein 18
Ciclev10003695m.v1.0	Ciclev10006151m.v1.0	Ciclev10022698m.v1.0	calmodulin 8
Ciclev10008863m.v1.0	Ciclev10009474m.v1.0	Ciclev10027336m.v1.0	aspartate-glutamate racemase family
Ciclev10011973m.v1.0	Ciclev10028664m.v1.0	Ciclev10031847m.v1.0	actin 1
Ciclev10010341m.v1.0	Ciclev10014255m.v1.0	Ciclev10014273m.v1.0	receptor kinase 3
Ciclev10003104m.v1.0	Ciclev10025354m.v1.0	Ciclev10030143m.v1.0	P-loop containing nucleoside triphosphate hydrolases superfamily protein
Ciclev10009423m.v1.0	Ciclev10009617m.v1.0	Ciclev10026493m.v1.0	no_ann
Ciclev10011400m.v1.0	Ciclev10028117m.v1.0	Ciclev10031134m.v1.0	Laccase/Diphenol oxidase family protein
Ciclev10001227m.v1.0	Ciclev10006312m.v1.0	Ciclev10020382m.v1.0	RING/U-box superfamily protein
Ciclev10000865m.v1.0	Ciclev10025414m.v1.0	Ciclev10027951m.v1.0	Protein kinase superfamily protein
Ciclev10005008m.v1.0	Ciclev10020558m.v1.0	Ciclev10023996m.v1.0	2-oxoglutarate (2OG) and Fe(II)-dependent oxygenase superfamily protein
Ciclev10009484m.v1.0	Ciclev10016276m.v1.0	Ciclev10016463m.v1.0	ethylene responsive element binding factor 4
Ciclev10012179m.v1.0	Ciclev10020412m.v1.0	Ciclev10031996m.v1.0	Peroxidase superfamily protein
Ciclev10003643m.v1.0	Ciclev10005469m.v1.0	Ciclev10023775m.v1.0	Seed maturation protein
Ciclev10021877m.v1.0	Ciclev10022253m.v1.0	Ciclev10022365m.v1.0	homeobox 7
Ciclev10012527m.v1.0	Ciclev10029155m.v1.0	Ciclev10032532m.v1.0	RNA-binding (RRM/RBD/RNP motifs) family protein
Ciclev10005072m.v1.0	Ciclev10020187m.v1.0	Ciclev10020257m.v1.0	UDP-D-glucuronate 4-epimerase 2
Ciclev10004816m.v1.0	Ciclev10014936m.v1.0	Ciclev10033633m.v1.0	MATE efflux family protein
Ciclev10002751m.v1.0	Ciclev10026642m.v1.0	Ciclev10029465m.v1.0	S phase kinase-associated protein 1
Ciclev10002236m.v1.0	Ciclev10002668m.v1.0	Ciclev10026333m.v1.0	Alba DNA/RNA-binding protein
Ciclev10017477m.v1.0	Ciclev10023449m.v1.0	Ciclev10032113m.v1.0	no_ann
Ciclev10012107m.v1.0	Ciclev10015933m.v1.0	Ciclev10032499m.v1.0	Clathrin light chain protein
Ciclev10011895m.v1.0	Ciclev10028567m.v1.0	Ciclev10031723m.v1.0	Protein kinase superfamily protein
Ciclev10005312m.v1.0	Ciclev10021116m.v1.0	Ciclev10021222m.v1.0	AT hook motif DNA-binding family protein
Ciclev10000126m.v1.0	Ciclev10006833m.v1.0	Ciclev10018645m.v1.0	ATPase E1-E2 type family protein / haloacid dehalogenase-like hydrolase family protein

Table S5. Assembly statistics of unmapped reads on the clementine genome for novel lineage-specific gene identification.

	sequenced read pairs			Assembly						Unique features		
Sample	raw	unmapped	Quality trimmed	Num. Scaffolds	assembled bases (bp)	N50	overlapping with Clementine (bp)	Recovered for annotation (bp)	gene products	scaffolds	Assembled bp	gene products
Citron	104,705,329	5,808,621	4,140,878	1,707	7,167,497	4,249	543,030	6,360,516	237	1,382	5,376,177	62
Pummelo	47,415,781	1,881,455	1,444,273	502	2,217,733	4,232	487,418	1,583,644	211	227	837,101	61
Tachibana	43,862,942	1,142,881	889,052	68	533,733	10,185	362,388	130,854	30	11	41,117	6
Sun Chu Sha	64,666,318	1,138,881	952,807	67	532,812	10,215	396,872	78,563	8	6	17,022	1
Clementine	122,428,729	1,757,153	1,311,084	283	1,174,311	3,804	N/A	N/A	N/A	N/A	N/A	N/A
